# Lumpy Skin Disease: A Systematic Review of Mode of Transmission, Risk of Emergence and Risk Entry Pathway

**DOI:** 10.3390/v15081622

**Published:** 2023-07-25

**Authors:** Juana Bianchini, Xavier Simons, Marie-France Humblet, Claude Saegerman

**Affiliations:** 1 Faculty of Veterinary Medicine, Research Unit in Epidemiology and Risk Analysis Applied to Veterinary Sciences (UREAR- ULiège), Fundamental and Applied Research for Animals & Health, (FARAH) Centre, Liège University, 4000 Liège, Belgium; juana.bianchini@uliege.be; 2 Unit Veterinary Epidemiology, Department Epidemiology and Public Health, Sciensano, 1050 Brussels, Belgium; xavier.simons@sciensano.be; 3 Department of Occupational Protection and Hygiene, Unit Biosafety, Biosecurity and Environmental Licences, Liège University, 4000 Liège, Belgium; mfhumblet@uliege.be

**Keywords:** lumpy skin disease, modes of transmission, vectors, stable fly, entry risk pathways

## Abstract

The spread of lumpy skin disease (LSD) to free countries over the last 10 years, particularly countries in Europe, Central and South East Asia, has highlighted the threat of emergence in new areas or re-emergence in countries that achieved eradication. This review aimed to identify studies on LSD epidemiology. A focus was made on hosts, modes of transmission and spread, risks of outbreaks and emergence in new areas. In order to summarize the research progress regarding the epidemiological characteristics of LSD virus over the last 40 years, the Preferred Reporting Items for Systematic reviews and Meta-Analyses statement guidelines were followed, via two databases, i.e., PubMed (biomedical literature) and Scopus (peer-reviewed literature including scientific journals, books, and conference proceedings). A total of 86 scientific articles were considered and classified according to the type of epidemiological study, i.e., experimental versus observational. The main findings and limitations of the retrieved articles were summarized: buffaloes are the main non-cattle hosts, the main transmission mode is mechanical, i.e., via blood-sucking vectors, and stable flies are the most competent vectors. Vectors are mainly responsible for a short-distance spread, while cattle trade spread the virus over long distances. Furthermore, vaccine-recombinant strains have emerged. In conclusion, controlling animal trade and insects in animal transport trucks are the most appropriate measures to limit or prevent LSD (re)emergence.

## 1. Introduction

Lumpy skin disease (LSD) is an emerging infectious disease of cattle and buffaloes which until recently had been considered as a neglected disease. First reported in Zambia in 1920, it spread to other African countries and became endemic in most sub-Saharan areas [1]. The disease was contained within this region until Egypt reported its first case in 1988 [1]. Then Israel experienced outbreaks in 1989 [2]. Between the 1990s and 2010, it was reported in countries of the Arabic peninsula, i.e., Kuwait in 1991, Lebanon in 1993, Yemen in 1995, United Arab Emirates in 2000, Bahrain in 2003, Israel (with recurring outbreaks in 2006 and 2007) and Oman in 2010 [3,4,5,6]. In 2012, Israel had another epidemic, and the disease reached Jordan and Iraq, followed by Turkey in 2013. Turkey is an important crossroad between Asia and Europe; in 2014, Azerbaijan and Iran reported their first cases, followed by Armenia, Greece and Russia a year later [6]. The spread continued towards Europe, and Georgia, Kazakhstan, Albania, Bulgaria, Montenegro, North Macedonia and Serbia reported outbreaks or cases in 2016 [6]. Certain countries, in particular European Member States, contained the outbreaks and no additional countries reported LSD cases during the 2017–2018 period. In 2019, LSD emerged in central Asia; China, Bangladesh and India reported their first cases during this year. Afterwards, it continued spreading in the center of Asia as Bhutan and Nepal reported their first cases in 2020 [6]. That same year, it also moved towards South-East Asia, i.e., Hong Kong, Myanmar, Sri Lanka and Vietnam. In 2021, LSD continued to be reported in new Asian countries, i.e., Mongolia, Pakistan and Taiwan, and continued spreading towards South-East Asia as Cambodia, Thailand and Malaysia reported their first cases. Finally, in 2022, Afghanistan and Indonesia reported their first cases [6]. 

Globalization, which has made changes in trading patterns of animals and animal products, global climate change and civil conflicts occurring in certain countries have aided the continuous spread of LSD virus (LSDV). LSD is a threat to livestock health and food security especially in lower income countries. These threats include important production losses, loss of draught power, reduced feed intake, disease management, trade restriction, and long-term convalescence. For this reason, it is listed as a notifiable disease in bovines by the World Organization for Animal Health (WOAH) [6].

These characteristics of the disease and several factors related to the evolving epidemiology of the disease raise a great concern in terms of introduction and difficulty of eradication, i.e., (i) non-stop and rapid spread towards South-East Asia, (ii) reoccurrence in countries where control and preventive measures had achieved eradication such as Russia, (iii) endemicity in previously free-countries such as Turkey and (iv) spread to regions experiencing a colder climate. Such concern has renewed scientific interest and a lot of new information on LSD epidemiology has appeared in the scientific literature. 

The aim of this literature review is to summarize the research progress regarding the epidemiological characteristics of LSDV over the last 40 years. It will analyze trends in the literature and the modes of transmission and spread, in order to establish the disease introduction pathway(s) and to assess the conditions of LSD (re)emergence. The final objective is to highlight future research directions that will contribute to the improvement of LSD prevention, control and eradication.

## 2. Materials and Methods

This systematic review was conducted in accordance with the Preferred Reporting Items for Systematic Reviews and Meta-Analyses statement (PRISMA) guidelines [7] (Appendix A). The literature search was performed on 1st of September 2022 in the PubMed (www.ncbi.nlm.nih.gov/pubmed (accessed on 1 September 2022)) and Scopus databases (www.scopus.com (accessed on 1 September 2022)), with the search term “Lumpy Skin Disease”. Only English-written articles, with an available abstract, and published between January 1980 and September 2022, were extracted. Editorials and books were excluded.

These articles investigated LSD hosts, transmission modes, risk factors of an outbreak and disease spread, as well as analysis of a risk of introduction into a new area. After excluding duplicates resulting from the search in two different databases, the remaining papers underwent a double-stage screening process, considering several inclusion and exclusion criteria, as shown in Table 1. The first exclusion criteria were applied to articles titles only, and the second exclusion criteria considered article titles and abstracts. Afterwards, articles were screened by reading them in full, and same second exclusion criteria were applied.

Articles included in this systematic review included different types of epidemiology studies. While some described certain characteristics of LSD epidemic, others focus on specifics of LSDV. Thus, in order to allow a proper analysis and create a better description of the articles, these were categorized according to the study design of study, i.e., experimental vs. observational (cross-sectional or descriptive), literature reviews, risk analysis of LSD introduction in a country. Afterwards, the following information was extracted and inserted into a summary table (see Appendix B): type of epidemiological study, methodology, modes of transmission, risk factors associated with LSD introduction/spread to a new location, vectors/wild animals involved, reservoir hosts, main conclusions and limitations of the studies.

## 3. Results

### 3.1. Selection Process

**Figure 1:**
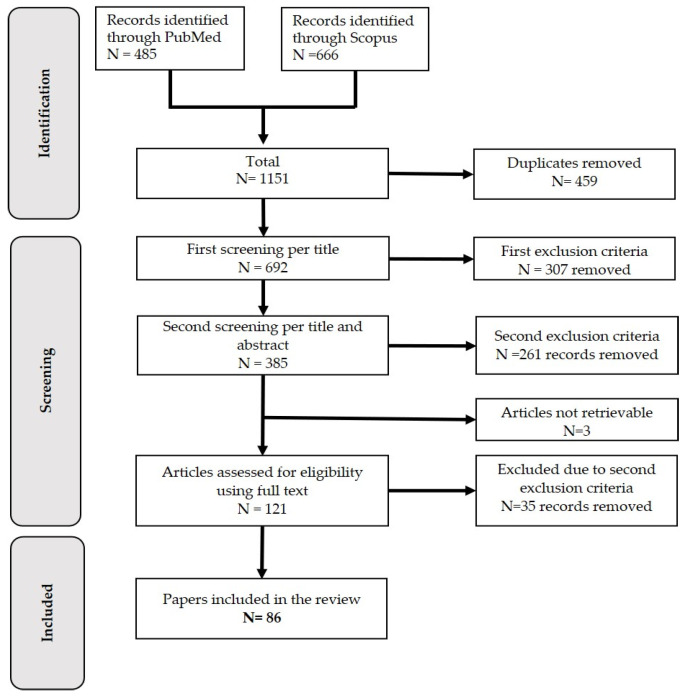
Flowchart representing the article selection process according to the PRISMA guidelines (N = 86).

The results of the selection process are shown in Figure 1. The search made in the scientific databases returned 692 articles after the removal of duplicates. By applying the first exclusion criteria only to the title of the articles a total of 385 articles were selected for the second screening process. In the second screening round a total of 261 articles were excluded based on secondary exclusion criteria applied to title and abstracts. A total of 124 were selected. The full text was accessible and read for 121 of them (three of the articles had to be excluded as their full text could not be accessed). From the articles read in full, 35 were excluded based on the secondary exclusion criteria. When there was doubt, a consensus meeting between the first and last author was held to decide on final exclusion. Finally, a total of 86 articles were included in the review. The full details of the reviewed articles are summarized in Appendix B.

### 3.2. Description of the Retrieved Articles

**Figure 2:**
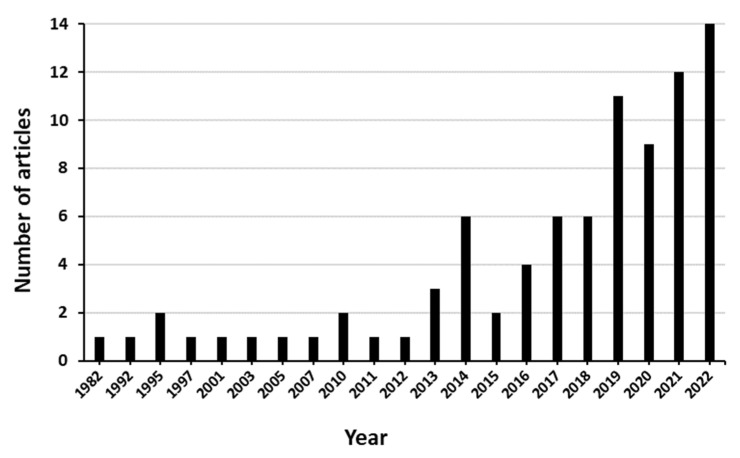
Number of articles per year (N = 86).

The frequency of publications shows that, between 1982 and 2010, only eight articles were published; in some years, there were no publications on LSD transmission or risk at all. After 2010, there was at least one article published per year, most of them being published afterwards (Figure 2). The highest number of publications was recorded in 2022 (N = 14), followed by 2021 and 2019, with N = 12 and N = 11 articles, respectively.

**Figure 3:**
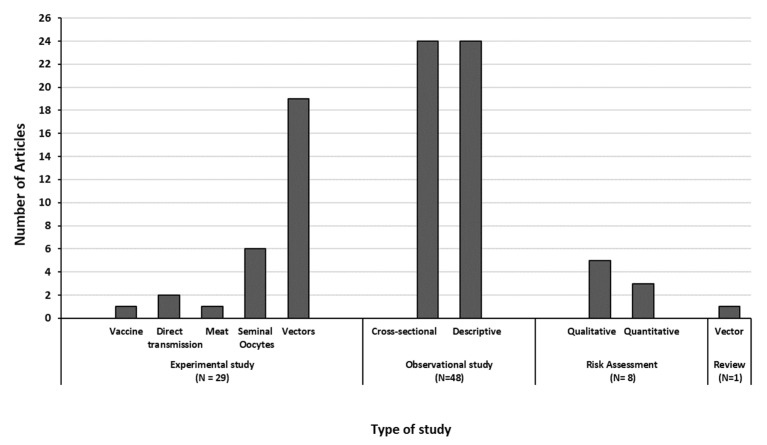
Categorization of articles selected by the screening process, according to the type of study (N = 86).

Based on the articles selected in the literature review process, the classification of studies, per category, is presented in Figure 3: most of them were observational studies, equally distributed between cross-sectional and descriptive studies. Experimental studies were mostly related with research on vectors. Only one literature review focusing on the role of *Stomoxys* flies in LSD transmission was included in this review. Table 2 shows the different studies and methodologies used in the selected articles.

### 3.3. Host of Lumpy Skin Disease Virus

Ten articles reported LSDV infection, via antibodies, clinical signs and PCR, in animals other than cattle (Table 3). Specifically, these animals were mainly free-ranging African buffaloes (*Syncerus caffer*) [62,63], which were classified as LSDV positive via serological test, and the Asian water buffalo [38,64,65,66]. Other reported African wild ruminant species with antibodies against Capripox viruses included an Arabian Oryx (*Oryx leucoryx*) in a wildlife reserve [67], southern elands (*Taurotragus oryx*) [69], Springboks (*Antidorcas marsupialis*) [69], Impalas (*Aepyceros melampus*) [69], and wildebeests (*Connachaetes gnou*, *C*. *taurinus*) (Table 3) [69]. The southern eland was also reported positive to LSDV by PCR [68]. A captive giraffe (*Giraffa Camelopardalis*) [70] was confirmed to be positive to LSDV by genomic detection and virus isolation in a Vietnamese zoo.

### 3.4. Modes of Transmission

#### 3.4.1. Direct Transmission

Direct transmission was investigated or reported in 12 articles: direct contacts between animals (N = 3) [34,35,52], seminal (N = 7) [28,29,30,31,32,33,72], intra-uterine transmission (N = 1) [71] and meat and offal (N = 1) [36].

The transmission via direct contact between animals was deemed as being ineffective. The 1995 experimental study [34] tested this route of transmission by performing seven separate experiments, in which one uninfected cow was housed in close contact with two infected animals for a month, in an insect-proof facility. The results showed that, although infected cattle excreted LSDV in saliva, nasal and ocular discharges, none of the healthy animals developed clinical signs or produced detectable levels of serum neutralizing antibodies (i.e., no infection occurred) [34]. In an Israeli study, mathematical modelling was applied to investigate three possible routes of transmission in a same herd: (i) indirect contacts between different groups in the same herd, (ii) direct contacts or contacts via common drinking water within each group and (iii) transmission by contact during milking. In that study, modelling was applied to data from an LSD outbreak reported in a dairy herd. In the presence of an infected cow, the basic reproduction number (R0) of indirect transmission was estimated at 15.7, compared to 0.36 for direct transmission. These results provided further evidence that indirect transmission was the only parameter that could solely explain the entire outbreak dynamics [52] and that indirect transmission is likely to be far more important than direct transmission.

However, a 2020 study [35] which conducted a similar experimental study established for the first time the transmission of LSDV between cattle via direct contact [35]. In that study, cattle were infected using a vaccine-derived virulent recombinant LSDV strain (Saratov/2017) and both infected and healthy animals were housed together for a 60 day-period, which means twice longer compared to the previous study.

Transmission of LSDV via bull semen was shown to be a possible route of transmission. Experimental studies highlighted that LSDV was present in semen from experimentally infected bulls and that bulls were positive to LSDV in all semen fractions, excreting the virus for prolonged periods (longer than 28 days) even when obvious clinical signs of the disease were no longer apparent [28,29]. Moreover, the virus has also been detected in the semen of naturally infected bulls [72]. The testis and epididymis were identified as sites of LSDV persistence [30]. Seminal transmission to uninfected heifers was reported [31]. Vaccination is effective in preventing the excretion of LSDV as the semen of vaccinated bulls tested negative to LSDV [28]. Regarding the presence of LSDV in cryopreserved semen and embryo production, experimental studies [32,33] showed that the virus could persist in semen even if it undergo standard treatments [33]; in vitro yield was significantly reduced by the presence of LSDV in frozen-thawed semen [32] with the resulting embryos testing positive to LSDV. Furthermore, when testing an LSD-infected herd, neutralizing antibodies were detected in a one-day old calf, providing evidence of intrauterine transmission [71].

Based on one single study the transmission through bovine meat and offal products would be very low [36]. Following experimental infection, it appeared that lymph nodes and testicles of clinically and sub-clinically infected animals were reservoirs of live LSDV whilst live virus was not detected in deep skeletal meat [36].

#### 3.4.2. Indirect Transmission via Vectors

The only route of indirect transmission retrieved in this literature review is via arthropod vectors. Twenty-nine articles focused on identifying possible vectors of LDS, and their potential role as mechanical (the vector simply “transport” the pathogen from one host to another), or biological vector (the pathogen undergoes replication and/or transformation inside the vector before transmission to other animals through subsequent blood meals) [93]. The identification of vectors potentially responsible of reported outbreaks was also assessed. These number of studies included 20 experimental studies (i.e., in laboratory conditions) [8,9,10,11,12,13,14,15,16,17,18,19,20,21,22,23,24,25,26,27], six observational studies [11,12,13,14,15,59,79] and one systematic review that focused on the role of stable flies [92]. There were four studies [18,19,39,66] in which the primary objective was not sampling LSDV from vectors in the field, but they were part of the study and thus included in the results. The groups of vectors cited in the selected articles were: the stable fly *Stomoxys calcitrans* (N = 12), mosquitoes (N = 6), biting midges *Culicoides* spp. (N = 6), ticks (N = 12), horse flies (N = 2) and non-biting flies (N = 2). Thus, two classes of arthropods were identified as potential vectors of LSDV, i.e., Insecta and Arachnida (ticks).

**Blood sucking vectors-Insects**

Experimental studies focused on establishing the competence and/or capacity of transmitting LSDV by different blood-feeding insect vectors. Parameters investigated for each vector are shown in Table 4.

Four experimental studies [8,9,10,11] assessed the potential role of stable flies, mosquitoes and biting midges as vectors of LSDV. These studies allowed comparing the different potential vectors. A first experimental study carried out in 2003 [8] intended to reproduce the mechanical transmission of LSDV by several blood-feeding insects, i.e., *Stomoxys calcitrans*, *Culex quinquefasciatus* and *Anopheles Stephensis* (mosquitoes), and *Culicoides nubeculosus*. The transmission attempt was made 24 h after feeding. None of the susceptible animals seroconverted or showed any reaction to exposure (i.e., no transmission was achieved). Furthermore, there was no evidence of viral replication in any of the aforementioned species. The virus was detected by PCR in *S. calcitrans* up to one day post-infective feed, only immediately post-feeding in *C*. *nubeculosus*, after 8 days in *Anopheles stephensis* and after 6 days in *Culex quinquefasciatus* [8]. 

Two studies [9,10] focused on *Stomoxys calcitrans*, *C*. *nubeculosus*, and mosquitoes *Culex quenquefasciatus* and *Aedes aegypti.* Authors quantified the acquisition and retention of LSDV in different anatomical locations of these species. Neither study included experimental transmission to healthy animals, and insects were not tested for the virus beyond 8 days post-infection. The probability of vectors acquiring LSDV from a subclinically infected animal was very low (0.006) compared with the probability of infection from an animal with clinical signs (0.23). An insect feeding on a sub-clinically-infected animal was 97% less likely to acquire LSDV than one feeding on a clinically affected animal. The probability of acquiring LSDV was substantially greater when feeding on a lesion compared with feeding on normal skin or blood from a clinically affected animals [9]. There was no evidence of virus replication in the vector and the mean duration of viral retention differed among the four insect species, being the longest for *Ae*. *aegypti* (5.9 days) and *S. calcitrans* (5.5 days), followed by *Cx*. *quinquefasciatus* (4.5 days) and *C. nubeculosus* (2.4 days) [9]. After feeding on a skin lesion, LSDV was retained on the proboscis for the longest period (mean duration: 6.4 to 7.9 days), followed by the head/thorax (5.2 to 6.4 days), and for the shortest time in the abdomen (2.1 to 3.3 days) [10].

The basic reproduction number (R0) for the same aforementioned species of insects was determined in two studies [9,11]. The first study published in 2019 [11] used a transmission model that considered the underlying process involved in the vector-borne transmission to cattle. The parameters included in the model were estimated by reanalyzing data from published transmission studies and using Bayesian methods to quantify uncertainty. Sensitivity analysis allowed for calculating R0 and determining the parameters with the greatest influence. The other study [9] used data from their quantification study, combined with data from the earlier study [11] to recalculate the R0 values. The results of both studies were relatively consistent, but the wide prediction intervals should be noted. The estimated R0s were the following: 19.1 (95% predictive interval of 2.73–57.03) [9] and 15.5 (95% prediction interval of 1.4–81.9) [11] for *S. calcitrans*; 7.4 (95% prediction interval of 1.3–17.6) [11] and 2.41 (95% credibility interval of 0.50–5.22) for *Ae. aegypti* [9]; 0.8 (95% predictive interval of 0.9–3.5) [11] and 0.55 (95% credibility interval of 0.06–2.37) for *Cx. quinquefasciatus* [9]; 1.8 (95% prediction interval of 0.06–13.5) [11] and 7.09 (95% credibility interval of 0.24–37.10) *C. nuberculosis* [9]. An R0 for *An. stephensis* was only estimated in the earlier study and reached 1.6 (95% predictive interval of 0.2–6.0) [11].

When considering all these studies [8,9,10,11], it appears that *S*. *calcitrans* is likely to be the vectors with most capacity of transmitting LSDV, as well as the mosquito species *Ae. aegypti*. By contrast, *C. nubeculosus, An. stephensi*, and *Cx. quinquefasciatus* are likely to be inefficient vectors of LSDV.

*Stomoxys calcitrans* was the most studied vector in the present review, through (i) four observational studies which investigated or inferred its role in LSD outbreaks [2,59,79,80], (ii) eight experimental studies that determined its vector competency [8,9,10,11,12,13,14,15] and (iii) one literature review that discussed its role in the LSD epidemic in the Russian Federation [92].

In field settings, *S. calcitrans* was suspected to be responsible for the first known LSD outbreak in 1989, in an Israeli dairy farm. Authors suggested that LSDV-infected *S*. *calcitrans* were carried by the winds from Egypt which was experiencing LSD outbreaks at that time. Such a hypothesis was based on the circumstantial evidence that there was no cattle trade with countries experiencing LSD outbreaks, strict control measures were implemented at the border and winds were adequate to carry infected *S. calcitrans* from Egypt [2]. A later work also performed in Israel [59] calculated the monthly relative abundance of each dipteran in each farm that had been affected by LSD 1–2 year previously. The relative abundances of *S. calcitrans* in the month parallel to the outbreaks (December and April) were significantly higher compared to other Diptera, and their populations peaked in the months of LSD onset in the studied farms. Using a stable fly population model based on weather parameters to validate these finds showed that the peaks in *S. calcitrans* numbers matched the peaks in monthly numbers of newly affected dairy farms in the study area. However, the observations and model predictions revealed a lower abundance of stable flies during October and November, when LSD affected adjacent grazing beef herds. Authors inferred that these results suggest that another vector was probably involved in LSDV transmission in grazing beef herds [59].

In 2021–2022, two observational field studies which sampled different blood sucking possible vectors [79,80] reported that LSDV was isolated from stable flies. In both studies the number of samples was very small; from an LSD outbreak in Kazakhstan only two *Stomoxis* flies were tested with just one being positive [79] and from sampling a south African feedlot out of the 53 samples collected, eight were positive [80].

The single literature review [92] used a compilation of information regarding the entomology of *Stomoxys calcitrans*, the spread of LSD of cattle in Russia in the years 2015–2019, and the climatic conditions of the regions where LSD cases were recorded. With this data reviewed in the study, the authors concluded that the peak incidence of infection occurred in the warm month indicating the significant role of the *Stomoxys* fly in the epidemiology of the disease, fitting the hypothesis that this fly was the culprit for the occurrence of LSD outbreaks. However, it was noted that there were cases registered of LSD during the autumn-winter period of Russia when the intensity of the *Stomoxys* was minimal or completely absent and some of the outbreaks occurred at distances longer than the fly’s flying ability. Thus, authors indicated that there were other factors that influence the spread of LSD in Russia during this period of study [92]. 

In experimental studies, LSDV was isolated from different body parts of *S. calcitrans*, but mostly from the proboscis [10,12]; the fly excretes the virus both by regurgitation and defecation [12]. No evidence of virus replication was found in the vector [8,12,13,14]. Additionally, transmission was successful when it occurred immediately [12,15], but not 24 h after feeding [8]. These findings suggest that the stable fly is a competent mechanical vector of LSDV. Furthermore, another experimental study demonstrated the incompetence of three *Stomoxys* spp., i.e., *S. calcitrans*, *S. sitiens* and *S*. *indica*, as biological vectors after inoculation with LSDV [12]. 

The role of mosquitoes was experimentally studied in six selected articles [8,9,10,11,16,17]. The mosquito species of concern were: *Ae. aegypti*, *Cx. quinquefasciatus*, *An. stephensi*, *Ae. japonicus*, and *Cx. pipiens*. All species were shown to harbor viable LSDV in their bodies for 4 to 10 days after oral exposure [8,9,10,16,17], although LSDV retention in *Ae. aegypti*, *Cx. quinquefasciatus* and *An. stephensi* varied among the studies. As previously mentioned, only *Ae. aegypti* was able to transmit LSD to susceptible cattle under experimental conditions [16]. Thus, retention of LSDV in mosquitoes might be a general feature but the mechanism remains unknown. All experimental studies reported that the mosquito acts as a mechanical vector, however the mode is not as simple as “dirty-pin” type of virus transfer.

The potential role of biting midges in the transmission of LSDV was investigated in six selected articles [8,9,10,11,17,39]. Four experimental studies focused on *C. nubeculosus* [8,9,10,17]. The transmission of LSDV to susceptible cattle by collected field *Culicoides* spp. and laboratory-reared *C. nubeculosus* could not be reproduced, although LSDV was detected in their body parts and virus was retained for some days [17]. Moreover, there was no evidence of virus replication in *C. nubeculosus* [8,9,10]. These studies concluded that biting midges are not competent mechanical vectors of LSDV. A single field study found that *C. punctatus* [39] collected from a Turkish outbreak were harboring LSDV, and authors suggested that it could play a role in the transmission of the virus.

Regarding the potential role of horseflies in the transmission of LSD, no pre-2019 publication was retrieved from this systematic literature review. One experimental study [15] tested the transmission of the virus to cattle by the horsefly species *Haematopota* spp. Transmission from infected to healthy animals was achieved. Authors established that their large mouthparts are in favor of mechanical transmission, as they can retain a high volume of blood, and thus inoculate higher viral doses during interrupted feeding on several hosts [15]. Finally, they suggested that horse flies could be more competent than the stable fly, since there were less of the former than the latter in the experiment.

Only one observational field study investigated the LSDV infection rate of horse flies: LSDV was isolated from 14.29% of horseflies *Tabanus bromiums* sampled during an LSD outbreak [79]. Although they could not confirm the transmission, the authors did not discard the potential implication of horseflies in the outbreak.

Non-biting flies have never been investigated experimentally, given that they have never been inferred as LSDV carriers. However, two recent observational studies [81,82] trapped different insects within the frameworks of surveillance campaigns after LSD outbreaks in Russia and in the West Chinese border; authors isolated LSDV DNA in *Musca domestica* and *Muscina stabulans*.

**Indirect transmission via ticks**

Thirteen articles [18,19,20,21,22,23,24,25,26,27,66,79,83], all of them published from 2011 onwards, investigated the vectorial capacity of hard ticks to be vectors of LSDV. Only five studies [18,19,66,79,83] sampled ticks obtained from the field. The authors of one study sampled ticks (species not specified) from LSD infected buffaloes, but the virus was not detected [66]. Within the frameworks of another field study that relied on the sampling of different vectors from an outbreak in Kazakhstan [79], authors isolated LSDV in four *Dermacentor marginatus* and nine *Hyalomma asiaticum* ticks. A single study used a large sample size of ticks (4000 adult ticks). Three pools of infected ticks out of 20 were found positive to LSDV, which extrapolates to 15% of the whole specimens were positively infected (i.e., 600 positive ticks) [83]. A study which obtained samples from both Egypt and South Africa found viral DNA in four out of four collected *Rhipicephalus* spp. from Egypt; and of the 52 samples collected from South Africa, 11 were *R*. *appendiculatus*, four *R. Boophilus*, seven *A*. *hebraeum*, four *H. truncatum*, two *Amblyomma* sp. and six *Rhipicephalus Boophilus* sp. [19].

From 2011 to 2015, experimental studies focused on the role of ticks as either mechanical or biological vectors of LSDV. Thus, the main focus of experimental studies (Table 5) was to determine if the tick would get infected after feeding (intrastadial infection), if it could persist in the tick’s life stages and progeny (transstadial, transovarial persistence), which tick stage(s) could infect an animal (transstadial, transovarial transmission).

The three tick species of interest were *Amblyomma hebraeum*, *Rhipicephalus appendiculatus*, and *R*. *decoloratus.* All the three species of ticks had intrastadial infection [20,26,27], transovarial passage and transmission [20,21,22,23,24,26,27]. Intrastadial transmission and transstadial persistence was demonstrated only by *A. hebraeum*, *R*. *appendiculatus* ticks [20,21,26,27]. An additional species which was investigated was *Rhipicephalus annulatus* [18]. These ticks were collected from cows in farms which were having LSD infections (i.e., naturally infected ticks) and incubated for oviposition to test the eggs and hatched larvae for the presence of LSDV. Thus, transovarial passage was observed [18].

One study [21] demonstrated the transstadial and transovarial transmission of LSDV by *A. hebraeum* nymphs and *R. decoloratus* female adults after a two-month exposure to night and daily temperatures of 5 °C and 20 °C, respectively, suggesting possible over-wintering of the virus in these ticks (i.e., possibility of these ticks being a reservoir for LSDV).

The latest study reported investigated the possibility of the tick being a biological vector. It attempted the in vitro growth of the virus in *Rhipicephalus* spp. tick cell lines and examined in vivo the presence of the virus in ticks collected from cattle during LSD outbreaks in Egypt and South Africa [19]. No evidence was obtained for replication of LSDV in tick cell lines although the virus was remarkably stable, remaining viable for 35 days at 28 °C in tick cell cultures, in growth medium used for tick cells and in phosphate buffered saline.

### 3.5. Emergence of Vaccine-Like Recombinant Strains

Between 2018 and 2022, nine articles [37,56,73,74,75,76,77,78,81,82] concluded that Russian and Chinese outbreaks were caused by a vaccine-like LSDV strain. For the first time, a vaccine-like strain (Neethling type) was identified during the 2017 Russian outbreak, in a region sharing a border with Kazakhstan [81]: the aforementioned strain was isolated in cattle and in house flies (*Musca domestica*) [81]. Although the route of introduction in Russia remains unclear, authors suggested that it was most likely due to the illegal use of the live attenuated homologous vaccines or the illegal movements of animals from Kazakhstan. It was highlighted in the Russian studies that while the use of homologous LSDV vaccines is not authorized in Russia, the Lumpivax vaccine (KEVEVAPI) was used in Kazakhstan shortly before the emergence of the vaccine-like strains [81]. This fortuitous finding led to a follow-up study on the epidemiological situation of LSD in Russia since 2016 [73]. The authors examined samples containing vaccine-like LSDV strains, collected in 2017 in the Privolzhsky Federal District, a Russian region that is geospatially outside the zone affected in 2016 and where live vaccines against LSDV had never been authorized or knowingly used. The study reported the widespread presence of vaccine-like LSDV strains in Russian cattle [73]. Following that first finding, sequential articles established the presence of vaccine-like strains. In 2018, the re-emergence of LSD was reported in Kurgan Oblast, Russia. The named ‘Kurgan/2018’ strain was neither from the vaccine nor from the field groups, strongly suggesting a novel recombinant profile [74]. In early March 2019, the Republic of Udmurtiya experienced an outbreak of LSD, while temperatures remain permanently below 0 °C, thus with no insect activity [75]. The causative LSDV (LSDV_Udmurtiya_Russia_2019) was shown to be a recombinant composed of a live attenuated Neethling-type vaccine strain (dominant parental strain) and a Kenyan KSGP/ NI-2490-like virus (minor parental strain) [75]. Furthermore, a recombinant vaccine-like LSDV from a 2019-outbreak in the Russian region of Saratov (Saratov/2019), where the first recombinant Saratov/2017 was documented, was described [76]. Even though both strains were isolated two years apart, Saratov/2019 seemed to be clonally derived from Russia/Saratov/2017, thus suggesting overwintering of the LSDV in the region since 2017.

A molecular epidemiology study conducted in Russia from 2015 to 2018 concluded that LSDV epidemiology had split into two independent waves. The 2015–2016 epidemic was attributable to a field isolate, whereas the 2017 epidemic, and in particular the 2018 epidemic, represented a disease importation, as the strain was not genetically linked [77]. A 2022 study analyzed the epidemiological evolution of LSD in Russia over a 6-year period, i.e., from 2015 to 2020 [56]. The results showed the disease tended to form spatiotemporal clusters in 2016–2018. These were associated with genetic changes in the virus and they were vaccine-like recombinant isolates; while the early clusters (2015–2106) were only formed by the field LSDV isolate [56]. Authors concluded that the LSD epidemiology could be affected severely by the use of homologous live-attenuated vaccines.

In 2019, China reported the isolation of a recombinant vaccine strain in the Xinjiang province, which borders Kazakhstan. That strain, named GD01/2020, was distinct from the two recombinant strains previously isolated in Russia [37]. Its origin remains unknown, but it was more probably introduced in the country in 2019 and responsible for the first outbreaks of that year, and eventually spread to other regions in the year 2022 [37]. This prompted to investigate insects as potential vectors involved and in 2022, a field study relying on the trapping of LSDV vectors was performed: the vaccine-like LSDV strain was isolated in two species of non-biting flies, i.e., *Musca domestica* L. and *Muscina stabulans* [82].

Given all the circumstantial evidence which pointed to the Lumpivax vaccine as the culprit of the emergence of these new recombinant vaccine strain, a study [78] analyzed the composition of two batches of the Lumpivax (KEVEVAPI) vaccine. Additionally, it investigated the possible link between the vaccine and the recent vaccine-like recombinant LSDV strains. By directly analyzing the genomes present in the vaccines they found that although labelled as a pure Neethling-based LSDV vaccine, the Lumpivax had a combination of at least three different *Capripoxvirus* strains: a Neethling-like vaccine strain, a Kenyan-like sheep and goat pox virus (KSGP) as well as an LSDV vaccine strain and a Sudan-like goatpox virus vaccine strain [78]. The genomic data of these finding indicated that the exchange of genetic material did not occur in co-infected animals but during vaccine production. The authors then concluded that the latest emergence of vaccine-like LSDV strains in a large part of Asia was therefore most likely the result of a spill-over from animals vaccinated with the Lumpivax vaccine which was poorly manufactured [78].

### 3.6. Risk Factors of Lumpy Skin Disease Outbreaks and Spread

Table 6 summarizes the selected cross-sectional studies (N = 17) which identified the main herd level risk factors for LSD cases and what geographic and climatic conditions are favorable to the disease occurrence and spread.

Farm level risk factors were assessed using multivariable logistic regression models in ten studies [38,40,41,42,43,44,45,46,47,48]. Table 7 summarizes the odds ratio (OR) obtained from such models. LSD positivity (i.e., outcome variable) was determined through blood sampling or clinical signs. There were different reported risk factors, being the three main reported risk factors (i.e., higher odds of presenting LSD): female cattle [41,42,46,47], animal movements (introduction of new cattle and sales) [40,43,44,45] and communal watering/grazing systems [40,42,43,45]. Other identified risk factors were genus and breeds (local breeds and buffaloes less likely to present LSD clinical signs) [38,40,47], and contact with other animals (sheep, goats, buffalo) [40,42,48]. Age and herd size showed different results as their group categories differed in the studies. One study showed a higher risk for medium and large size herd [46], and another the contrary [44]. Likewise, age showed various results, young cows had higher risk [38,46] and in others older ones were at risk [40,42,48]. Two studies which included weather conditions in their models found that higher risk was found the summer season [40], and a mean annual rainfall of 1001–1200 mm [42].

Three studies used ecological niche modelling to investigate the association between environmental factors (e.g., climate and land cover) and location data on disease outbreaks [49,50,51]. These associations were then used to predict the geographic distribution of LSDV in underreporting regions. Two of those studies focused on used land geography, not borders [49,50], thus including several countries, while the other used data from an Iranian region [51]. These studies concluded that environmental predictors contributing to the ecological niche of LSDV were: annual rainfalls, land cover, higher mean diurnal temperature range, type of livestock production system and global livestock densities. One study [49] identified wind speed as an important driver explaining the observed distribution of LSDV; higher wind speeds were negatively associated with LSDV incidence.

Another study used spatial regression model to predict the risk of LSD spread in neighboring free-countries of Europe and Central Asia [57]. They reported a significant effect of land cover, cattle density of the area, as well as higher annual mean temperature and higher mean diurnal temperature range on the occurrence of an LSD outbreak [57]. Using time series analysis and spatial distribution to detect seasonality and cyclical patterns in LSD outbreaks reported that LSD incidences were registered in warm and humid highlands [55]. Likewise, when analyzing the LSD epidemic from 2015 to 2020 in Russia the seasonality of LSD for that period showed that outbreaks occurred during warm months between May and October with the highest peak of incidence in July. It also reported cases in November 2018 and March 2019 when there were winter conditions (snow and freezing temperatures) [56]. It also showed that the distribution of outbreaks tended to occur at higher levels in backyard cattle compared to commercial farms [56]. 

A study using mathematical models [53] reported the daily transmission rate between animals was slightly lower in the crop–livestock production system (0.072; 95% CI 0.068–0.076) compared to an intensive production system 0.076 (95% CI 0.068–0.085) [53]. Similarly, a 1.07 R0 (95% CI 1.01–1.13) was estimated between animals in the crop–livestock production system (95% CI 1.01–1.13), vs. 1.09 between animals in the intensive production system (95% CI 0.97–1.22) [53]. 

Regarding the spread modalities, the studies included in this literature review [54,58,60,69] reported that short-distance spread (i.e., between herds) was most likely attributed to a dispersal by arthropod vectors, whereas long-distance spread (i.e., transboundary, introduction into new geographical areas) was related to livestock movements. Both short- and long-distance spreads are associated with climatic conditions, especially a high temperatures and rainfalls. A study performed in the Balkans suggested that LSD was mostly transmitted at a rate of about 7.4 km/week and was due to a local, vector-borne spread [54]. However, a faster transmission at longer ranges, i.e., around 54.6 km/week, which is less frequent, was attributed to movements of infected animals [69]. Another study used a Kernel-based approach to describe the transmission of LSDV between herds in Albania [60]. All transmission routes were combined in a single generic mechanism with the probability of transmission from an infected to a non-infected herd assumed to depend on the distance between them (i.e., transmission). The authors inferred that transmission occurred over <5 km distances, which can be attributed to vectors, but with an appreciable probability of transmission over longer distances, that can be related to livestock movements [60]. Spatio-temporal analysis of LSD outbreaks that affected dairy farms in north-eastern Thailand discovered that these outbreaks occurred in numerous dairy farms over a short period of time, and that several affected farms were concentrated in the area [58]. Based on these findings and on the fact that cattle movements between dairy farms are few, the spread was attributable to vectors. A geographic information system (GIS) software [41] concluded that the introduction of the disease in Turkey may have originated from Syria and Iraq, as movements of live animals are reported across the Syria–Iraq border; furthermore, the first outbreak was recorded near the border.

Another climatic factor that has been under consideration of long-distance spread by carrying infected vectors is winds. Following the previous study [2] which proposed the hypothesis that the first LSD outbreak in Israel was most likely caused by the *Stomoxys* carried from winds of Egypt, Klausner et al. 2017 [61] identified relevant synoptic systems that could have allowed long-distance dispersal of infected vectors by wind from Egypt to Israel in the month preceding the 1989 and 2006 outbreaks [61]. However, this is conditioned by the vector’s survival.

### 3.7. Risk Analysis of Introduction of Lumpy Skin Disease to a Free-Area

Eight studies assessed the risk of LSD introduction in a country, i.e., five qualitative [84,85,86,87,88] and three quantitative risk assessments [89,90,91] (Table 2). With the exception of one study [87] conducted in Turkey, all risk analyses related to importation were performed in three historically LSD-free European countries, i.e., United Kingdom [84,85], Ukraine [86] and France [88,89,90].

All qualitative assessments [84,85,86,87,88] determined that the risk of introduction and/or spread of LSD in a country by pathways others than animal movements or vector transmission (excluding the tick) was “negligible”. Although considered slightly higher, the risk of introduction via animal movements or arthropod vectors (excluding the tick), was still estimated as “low”.

As LSD is endemic in Turkey [87], the following risk question was raised: “What is the probability of cattle LSD being introduced in the animal market?” Based on different release scenarios, the risk was considered as “high”. In the overall exposure assessment, the authors considered two different pathways, i.e., the probability of cattle being exposed to LSDV during seasonal migration—risk considered as “high”—and the probability of exposing cattle to LSDV from veterinary equipment—risk considered as “medium” [87].

Regarding the quantitative approach, stochastic models assessed the risk of LSD introduction in France [89,90]. One study considered the risk of introduction by arthropod vectors through animal transport trucks [89]. The annual risk of LSDV being introduced by *St. calcitrans* travelling in animal trucks was between 6 × 10^−5^ and 5.93 × 10^−3^ (median: 89.9 × 10^−5^); it was mainly related to the risk that insects transported in vehicles come from high-risk areas to enter French farms. The risk associated with the transport of cattle to slaughterhouses or horse transport was much lower (between 2 × 10^−7^ and 3.73 × 10^−5^, and between 5 × 10^−10^ and 3.95 × 10^−8^, for cattle and horses, respectively). The other risk analysis [90] focused on the importation of cattle in France. Authors estimated that the probability of the first LSD outbreak to occur after importation of infected live cattle for breeding or fattening was 5.4 × 10^−4^ (95% probability interval (PI): 0.4 × 10^−4^; 28.7 × 10^−4^) in the summer and 1.8 × 10^−4^ (95% PI 0.14 × 10^−4^; 15 × 10^−4^) in the winter [90].

**Figure 4:**
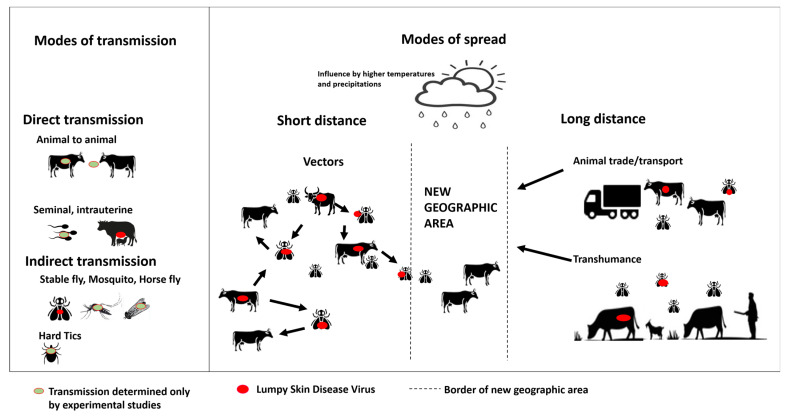
Diagram illustrating modes of transmission and spread of lumpy skin disease.

A generic framework for spatial quantitative risk assessments of infectious disease used LSD as a case study. Such an approach was carried out to assess the risk of LSDV spreading to other European countries after its introduction in the Balkans, in 2016 [91]. One single pathway of introduction was considered, i.e., registered movements of cattle: the highest mean probability of infection was in Croatia, followed by Italy, Hungary and Spain. Figure 4 illustrates a summary of the main modes transmission and spread which were established in this literature review.

## 4. Discussion

The aim of this paper was to review the general epidemiological characteristics of LSD described over the last 40 years in order to better understand the continuous emergence and spread of LSD to new areas. Unlike other reviews, which have usually focused on specific aspects of the disease in determined locations/regions, this systematic review is the first that aimed to cover aspects of epidemiological data related specifically to LSD modes of transmission, pathways of introductions and conditions of (re)emergence.

During the last 5 years, the research on LSD modes of transmission and risk factors or areas at risk of an outbreak has substantially increased, which confirms that the disease is becoming a global concern. Such increased interest is correlated to the arrival of LSD in Eastern Europe, Russia and Asia. The methodologies used have also evolved, as analyses have focused on finding additional LSD vectors and on geographical niches suitable for LSD to become endemic.

LSDV is host-restricted, similarly to other viruses of the genus Capripoxvirus. Although diagnosis of LSDV was performed mostly by serological methods, of which the main limitation is the lack of distinction between all Capripoxviruses, it is safe to assume that besides cattle, the other affected species are African and Asian water buffaloes, and just a few additional wild ruminant species [62,63,64,65,66,67,68,69,70]. Buffaloes seem to be more resistant to the disease than domestic cattle as studies reported less seropositivity, although it should be considered that the number of tested samples which studies reported were usually small. It was also suggested that the African buffalo could maintain the LSDV during non-epidemic periods [63]. This inference however was made only on the basis of positive samples with no additional information given to the context of when the samples were taken (i.e., time of year, other LSD outbreaks in the area). Thus, the role of the buffalo in the epidemic of LSD still remains to be elucidated. Indeed, to date, no experimental infection has been conducted in buffaloes, to establish the clinical signs or viraemic periods. This is of particular importance as, in some countries, buffaloes live close to or are part of the herd; they could represent a source of LSD infection in cattle herds. Moreover, they live also in countries which are still LSD-free, so their infection might go unnoticed until an outbreak occurs in cattle. Thus, understanding the biology of LSDV with the buffaloes would give a better insight of its role in the epidemiology of LSD.

In this review, little evidence was reported regarding the role of other wild ruminant species as LSDV hosts or sources of outbreaks. This is expected as studies on wildlife prevalence require economic and human power resources. All but two studies [68,70] reported wild animals positive to LSDV using serological testing. Although they reported them as LSD positive, this type of testing has the main limitation that current serological tests for LSDV cannot differentiate antibodies (Abs) to the virus from Abs towards other Capripoxviridae, i.e., Sheeppox virus and goatpox virus. Thus, it cannot be known with certainty that it was the LSDV causing the immunology response. Another important consideration is that animals with a mild or asymptomatic LSDV infection do not always develop a level of Abs detectable by a neutralization assay. Additionally, serological positivity does not necessarily imply that the virus replicates in the animals and that there is excretion; thus, they may not be able to transmit the virus. This could explain why clinical signs were only reported in one captive Arabian Oryx [67] and one giraffe [70]. Wild animals showing clinical signs of LSD are likely to be more susceptible to predators, which could explain the lack of reports of clinical disease in wild species. In addition, the presence of LSD clinical signs in wildlife might be easily missed, as the monitoring of skin lesions is difficult or impossible in their geographical settings. With all these considerations taken into account, it could be possible that the actual number of LSDV-infected wild ruminants may be considerably higher. Regardless of the difficulties mentioned, studies on LSD prevalence in wildlife should be encouraged as the virus may affect other Asian or European wild life, particularly those of the Bovidae family such as the European bison (*Bison bonasus*). Indeed, if LSD is introduced in a new geographical area where different wild ruminants coexist (either farmed or free ranging) and are naïve to LSDV, they could be infected transmit and maintain the disease. This could modify the dynamics of LSD epidemiology, making future outbreaks harder to control.

Regarding the modes of transmission, evidence from studies included in this literature review shows that direct or indirect transmission without the intervention of vectors is ineffective. The latest study that tested this route [35] managed to achieve a direct transmission between animals. Although there were important differences compared to the previous study [34] (virulent recombinant field strain and longer period of co-housing), such a finding highlights the importance of establishing further studies on LSDV biology. It is a priority to gain insights into whether the transmission achieved in this study is a de novo-created feature absent from both parental strains of the novel (recombinant) LSDV isolate used, or whether it was dormant but unlocked after genetic recombination. The study [52] which used mathematical modelling to estimate parameters of transmission modes also established that direct transmission was unlikely. However, the data used in the latter study came from an Israeli LSD outbreak in which all animals showing severe clinical signs were removed from the herd immediately, which may have artificially reduced the consequences of animal-to-animal contact.

Regarding other modes of direct transmission, the only plausible mode seems to be via seminal pathway. Experimental studies showed that LSDV is present in semen and seminal transmission was also achieved [28,29,30,32,33,72]. LSDV was detected in frozen semen samples which were collected from naturally infected bulls [31]. However, the effectiveness of such mode of transmission in the field still needs to be assessed. Given that laboratory conditions are controlled (e.g., infection of bulls with a virulent LSDV strain, the sample being collected during the viraemic period), the scenario differs from that which occurs in the field. The same comment is worth making for intrauterine transmission as a report included in this literature review mentioned that one single calf was considered as LSD-positive based on neutralizing Abs concentration [71]. It is unknown at what stage of pregnancy the cow was infected by the virus, and only one single calf was considered. Thus, these routes are still considered as unimportant when considering the spread of LSDV into new geographic areas (in contrast to other viruses of the genus Capripoxvirus, i.e., sheep and poxviruses in which direct contact or via aerosol are important).

Mechanical indirect vector-borne transmission is still considered as the main mode of transmission of LSDV, thus vector capacity and competence were extensively investigated, both in experimental and field studies. It important to distinguish the terms “vectorial capacity” and “vector competence”. Vectorial capacity is a measure of the transmission potential of a vector borne pathogen within a susceptible population. Vector competence, a component of the vectorial capacity equation, is the ability of an arthropod to transmit an infectious agent following exposure to that agent [94]. This distinction was not always made in the articles retrieved in this literature review as these terms are often used interchangeably to describe the ability of a vector to transmit a disease. Although this distinction was not always clarified in the research articles, it can be concluded that experimental studies focused mainly on the competence of hematophagous insects and hard ticks. Regarding vector competence, it is safe to assume that from the tested vector the stable fly *Stomoxys* spp. is the most competent vector of LSD as it could transmit LSDV in more than one of the experiments and presented the longest LSDV harboring time [8,9,10,11]. Given that it is the vector with the highest competency, it is also the vector with the highest vectorial capacity, as some observational descriptive and cross-sectional studies and the literature review determined they were the most abundant and inferred as the culprit of LSD outbreaks [2,59,79,80,92].

Moreover, studies reported it with having the highest R0 within the blood sucking insects studied (i.e., stable fly *Stomoxys calcitrans*, mosquitoes *Ae. Aegypti*, *Cx*. *quinquefasciatus*, *C. nubeculosus*) [9,11]. Furthermore, this insect is ideally suited to this type of virus transmission as it has a painful bite, which results in animals taking defensive actions such as tail switching, thus preventing the completion of a full blood-meal (i.e., interrupted feeding) and moving into the next animal [8]. This characteristic increases their vectorial capacity. 

Given their importance as biological vectors in several diseases, mosquito species were among the blood sucking insects studied in experimental conditions. From the species studied, *Ae. aegypti* seems to be the most probable competent as it harbored the virus for the longest period [9], presented the highest R0 among the three mosquito species [9,11] and was shown to be fully capable of LSDV mechanical transmission [16]. By contrast, *An. stephensi*, and *Cx*. *quinquefasciatus* are more likely to be inefficient vectors of LSDV. However, considering that, on one side, in laboratory experiments, mosquitoes are fed via spiked blood through artificial membranes or cotton pads soaked in blood spiked with LSDV and, on the other side, its anthropophilic character (not relevant in a farm environment), its capacity as an LSD vector is mostly likely reduced in natural field conditions.

The biting midges have been proposed as vectors for LSD as they play a major role in the spread of other important ruminant pathogens, i.e., Bluetongue and Schmallenberg virus. However, the results show that they should be considered as incompetent vectors for LSD. Indeed, under laboratory conditions, *C. nubeculosus* was not able to transmit the virus to susceptible animals, no viral replication was observed and they were already negative to LSDV 24 h post-feeding [8,9,10,17]. Given its poor vector competency, although the virus was isolated from *C. punctatus* collected on infected farms [39], it is probable that its capacity to transmit the disease is low.

Until recently, there was no direct evidence of the role of tabanids in the transmission of LSDV although they are able to mechanically transmit a wide range of pathogens (e.g., *Trypanosoma evansi*, *Besnoitia besnoiti*) and are regularly found around cattle. A recent study achieved the transmission of LSDV by tabanids, and even inferred that they could be more efficient than stable flies in transmitting the virus, given their large mouth. Thus, tabanids could be competent mechanical vectors. Given that this was the only experimental study which used tabanids [15] and only a single study reported LSDV in field collected tabanid [79], their vector capacity is not clear. However, they are contained to outdoor cattle and do not enter buildings or vehicles, if a horse fly enters a truck, it rapidly wrecks its wings, loses its flying ability and dies within a few hours [89]. Thus, more experimental and field studies focusing on tabanids are necessary to establish their role in transmitting and spread LSDV (i.e., evaluate its vectorial capacity).

The role of the non-biting flies *Musca domestica* and *Muscina stabulans* in the LSD epidemic only until recently came under questioning when DNA of LSDV was isolated in the aforementioned flies collected in new LSD outbreaks in Russia (2019) [81] and China (2020) [82]. Such an observation raises questions on whether they had been the culprits of introducing LSD in these new areas, as these flies are well-known mechanical vectors of numerous viruses and bacteria and feed off ocular discharges and skin lesions [95]. Further competence and surveillance studies on non-biting flies are necessary in order to establish their eventual role in the transmission and spread of LSD.

Ticks transmit several viruses, e.g., *Flaviviridae*, that cause encephalitis-like diseases (e.g., tick-borne encephalitis virus, Kumlinge virus and louping ill virus), and *Bunyaviridae*, responsible of hemorrhagic fevers (e.g., Nairobi sheep disease virus and Crimean-Congo hemorrhagic fever virus). Thus, the role of ticks as biological vectors of LSDV has always been of interest. The results of this systematic review show that only hard ticks were associated with LSDV transmission [18,19,20,21,22,23,24,25,26,27,79,83]. However, their role in outbreaks or epidemics is not clear. In this systematic review, only four field studies sampled ticks in search of LSDV [19,66,79,83], so the virus infection rate remains unknown in ticks. Experimental studies focused on the competency of ticks to act as biological vectors, and as such, to be reservoirs of the virus, and transmit it to their progeny and to recipient cattle [18,19,20,21,22,23,24,25,26,27,83]. Experimental studies achieved mechanical intrastadial, transstadial and transovarial transmission of the virus in both *A. hebraeum* and *Rh. appendiculatus* tick species, under cold temperatures. Although the passage of the LSDV between tick stages was achieved, studies could not establish that the tick could act as a biological vectors. Studies only determined mechanical transmission. As for their role in the epidemiology of LSD, ticks remain attached to the host for a long period, and thus one could discard their responsibility in a rapidly spreading epidemic. It is more likely that, if ticks are involved in the disease epidemiology, they act as a reservoir of the virus, and possibly maintain it during cold seasons. This may explain the capacity of the virus to overwinter outside the arthropod period of activity which has been reported in Russia [76].

As for the modes of spread, these are associated with modes of transmission. Risk factors studies at a herd level (i.e., short distance spread) using logistic regression [38,40,41,42,43,44,45,46,47,48], had differences on how they defined a herd or animal as being positive to LSDV. Some studies relied on serological tests (ELISA) while others considered LSD clinical signs reported by the cattle holder or veterinary services to consider if an animal or herd positive to LSD. This may affect the number of positive animals as it could be under- or overestimated. Indeed, serological tests could give false positive results (cows may develop Abs after exposure to sheep and goat poxviruses). On the other hand, the reliability of a person observing clinical signs depends on his/her knowledge and ability to clinically diagnose LSD. Additionally, the sample size and strategy were not systematically conducted and/or reported. The chosen risk factors to be considered in the logistic regression model varied among the studies; indeed, some studies lacked important variables (risk factors) such as climate, geographical location and herd vaccination status. Despite these important differences and the geographical diversity of study locations, three herd risk factors were consistent. Cattle trade, i.e., purchases, sales, introduction of new animals in the herd, increased the risk of LSD prevalence in the herd. Females are more likely to develop LSD than males, as it is the case for foreign breeds compared to buffaloes and local breeds. As for the breed of cattle, studies in endemic countries reported that local breeds of dairy cattle, i.e., *Bos indicus*, may present some natural resistance to the virus compared to foreign breeds such as Holstein cattle [39,40,47]. Although these results need to be taken with caution given their differences in methodology, it is important to take them into account as many countries that are currently experiencing or reporting new outbreaks of LSD (e.g., Thailand, Indonesia) may have herds mainly composed of foreign breeds, which could lead to higher number of cases and more outbreaks over time. Other mentioned risk factors were directly related to herd management, such as the sharing of pastures and water sources. Although three studies reported these factors as having a higher risk [40,42,43,45], they did not specify how the sharing was organized, e.g., shared among different farms or shared by the same herd. Consequently, another study reported that fenced farms were at higher risk of reporting LSD compared with farms sharing pastures [42]. Age and herd size was a risk factor included in most of these studies [38,40,41,42,43,44,46,48]. However, each study categorized them with different cut-offs. Thus, results differed and the effect of age and herd size on risk for presenting LSD cannot be determined. 

All studies agreed that blood-feeding insects are responsible for short-distance spread while long-distance spreads are related to animal movements. The spread through blood-feeding vectors is also conditioned by climatic conditions: indeed, higher temperatures and rainfalls are correlated with a higher vector activity, and thus the risk of outbreak increases [40,41,42,43,44,45,46,47,48,49,56]. Field studies supported that statement: most LSD outbreaks occur in the summer, after the rainy season, by the time of peak arthropod activity. Animal movements, via legal or illegal transports, are associated with long-distance spread. Additionally, the risk analyses included in this systematic review showed that animal transport, along with the vector-borne character, pose the highest risk of LSD introduction in a country [84,85,86,87,88,89,90,91]. Although these studies each have their own limitation (Appendix B), it safe to establish that animal trucks can transport not only cattle, but vectors as well. The spread is also conditioned by the geographic origin of animals and the duration of transport (with or without interruption). It is also important to consider that the control of transboundary animal movements (higher transhumance) is lacking in low income or politically unstable countries (conditions which pose difficulties to include when formulating a risk analysis of introduction model) which favor the illegal or uncontrolled movement of cattle. Other modes of spread, such as the trade of animal products or sub-products, are not a viable mode for LSDV, given the results of experimental studies [36]. Indeed, the qualitative risk analysis always deemed this route as ‘null’ [84,85,86,88].

As for the conditions favoring the (re)emergence of LSD, studies based on different modelling methods showed seasonality as an influence factor. Indeed, the risk is positively associated with higher diurnal/annual mean temperatures and annual rainfalls, i.e., geographical areas experiencing a humid and warmer weather are more at risk of emergence of LSD [49,50,51,53]. Geographical areas with higher cattle density were reported of being at higher risk of LSD occurrence [57]. Likewise, global livestock densities were one of the most important environmental predictors that contributed to the ecological niche of LSDV [50]. The type of livestock production system was also considered an environmental predictor when using this type of model [50]. Additionally, the daily transmission rate (R0) between animal was found to be slightly higher in intensive production systems [53] than in crop-livestock production systems, although the differences reported in this study were insignificant. Regardless of the differences in type of epidemiological model used, these results show that higher number of livestock and concentrated in an area pose a risk for emergence of LSD. This is most likely related to the reason of the mode of transmission of LSD, i.e., a higher concentration of livestock is correlated with a higher number of vectors.

This systematic review showed that novel vaccine-like strains have emerged and were responsible of some LSD outbreaks in Russia and China [56,73,74,75,76,77,81,82]. This has raised concerns as the reversion to virulence of a strain included in a live inactivated vaccine has been previously cited in the case of bluetongue vaccination in Europe (e.g., [96]). However, the emergence of this vaccine-like strain in Russia was most likely due to a poorly manufactured Lumpivax vaccine (KEVEVAPI) [37], which was widely used in neighboring Kazakhstan. Nevertheless, since these first reports, the epidemiological situation has become more complicated, as some countries such as Vietnam, Thailand and Mongolia reported that the newly emerged outbreaks were not caused only by field strains but also by novel recombinant vaccine-like strains. Thus, these newly emerged strains have spread to other countries and the effects on the epidemiology of LSDV are yet to be elucidated. Given that vaccination is the most efficient way to control and eradicate the disease, with successful examples in the Balkan region and Israel and emergency situations warrant their use, regulatory measures concerning vaccine manufacturing need to be implemented with strict rigorous controls and vaccination campaigns to be conducted using proper protocols.

The transmission of LSDV by contaminated needles used during vaccination campaigns has been suggested as a potential mechanism for the spread of infection within a herd [97]. However, no study retrieved in this literature review reported this mode as a risk factor, and thus it could be safely said that the risk is very low.

The spread through blood-feeding vectors is also influenced by climatic conditions: indeed, higher temperatures and rainfalls are correlated with a higher vector activity, and thus the risk of outbreak increases [40,41,42,43,44,45,46,47,48,49,56]. Another climatic condition that needs to be highlighted is winds. Long distance spread of LSDV-infected vectors carried by winds started to raise concern when Israel experienced outbreaks in 1989 and 2006. The author of this theory concluded that although it is a viable route, it depends of the vector’s capacity [61]. Given that there are some examples of possible transmission of other viruses through wind-assisted travel of vectors, e.g., it was proposed that Japanese encephalitis virus was introduced to Australia by wind-blown *Culex* spp. [98], and wind assisted in the spread of bluetongue virus in Europe [99], this route merits further investigation as LSD could reach countries by crossing geographical areas in which animal trade is easier to control (e.g., an island). Moreover, a study using ecological niche models to quantify the potential distribution of pathogens by correlating environmental abiotic conditions (e.g., temperature, precipitation and wind speed) with disease occurrence location, determined that wind speed was negatively associated with LSDV incidence [50]. Thus, wind is a climatic condition that may have effects on the epidemiology of LSD, but confirmation is needed.

In summary, the most efficient pathways for the emergence of LSDV in a country are the introduction of infected animals (in particular for long-distance spread) and the active transport of flying vectors to a naïve country (short-distance spread, e.g., from infected areas close to the borders). The risk of emergence is conditioned by: (i) climatic factors, i.e., warm weather promotes a higher vector activity and thus increases the risk of emergence, (ii) adverse economic situation, as border control is lacking, (iii) illegal or uncontrolled cattle movements, (iv) poor disinfection practices, (v) small cattle holdings and (vi) the use of poorly manufactured vaccines.

## 5. Conclusions

In conclusion, this systematic review reveals an increasing number of studies in countries where the disease is not endemic yet. Modelling LSD field data has become more specific and complex, thus broadening the epidemiological knowledge on the disease. Additionally, biotechnology has also advanced and research does not rely only on serology to confirm the diagnosis of LSD. Field and experimental studies have shifted towards the investigation of vectors others than stable flies. These conditions are positive, as the ultimate goal is to understand LSD epidemiology and stop its introduction in free-countries. The emergence in the Balkans, Europe, and Russia, where outbreaks are still reported, have required the rapid implementation of vaccination campaigns to control disease outbreaks and prevent its further spread. Indeed, vaccination is the only effective control and preventive strategy and remains the main approach to protect animal health and prevent economic losses. However, when considering the vaccine-associated outbreaks, there is a need to improve vaccine manufacturing standards, and to ensure quality control and traceability. Recent findings, i.e., new potential vectors, LSDV overwintering and new vaccine-recombinant strains, illustrate the multiple gaps in understanding the epidemiology, genetic features and transmission mechanisms of LSDV, which significantly impede the development of control strategies. A better understanding of LSDV will improve control programs in newly infected but also endemic countries. Insect control in cattle herds and transport vehicles is a crucial measure to prevent the emergence of LSD. Vaccination campaigns immediately after the emergence in a free country are easier to implement in high-income countries. In low-income areas, mitigation measures such as farmer education to detect LSD clinical signs, so they can identify the disease and notify the authorities, and insect control should be encouraged, along with vaccination during the period of vector activity. The control of LSD in endemic countries will reduce the risk of introduction and spread in neighboring nations.

## Figures and Tables

**Table 1:** Inclusion and exclusion criteria for peer-reviewed studies included in this review.

Inclusion Criteria	Articles published from 1980 to September 2022
	Studies focused on epidemiological characteristics of LSDV (i.e., hosts, animal reservoirs, vectors)
	Studies reporting LSD modes of transmission
	Studies analyzing historical or new outbreaks data with the purpose to highlight LSD risk factors
	Studies describing quantitative and/or qualitative risk modelling of LSD
	Studies reporting LSDV in ruminants other than cattle
Exclusion Criteria	*First exclusion criteria*
	Editorials, letters to the editor
	Studies related to a pathogen other than LSDV
	Studies concerning the investigation of LSDV molecular characteristic
	Studies on surveillance of LSDV
	*Second exclusion criteria*
	Articles describing modelling of economic impacts of LSD
	Studies reporting vaccine efficiency, molecular interaction of LSD, or LSDV characteristics
	Studies to evaluate test performance or surveillance systems
	Studies on outbreak control
	Reports on clinical signs
	Studies focusing on the prevalence of LSD and excluding its transmission and the risk factors of outbreaks
	General literature reviews of LSD

Legend: LSD, lumpy skin disease; LSDV, lumpy skin disease virus.

**Table 2:** Type, methodology and objective of the study from the articles retrieved in this systematic literature review.

Type of Study	Methodology	Objective of the Study	Count	References
Experimental studies	Experimental infections Molecular techniques to detected LSD. PCR, neutralization, gene sequencing	Vector competence of blood-sucking insects/ticks.	20	[8,9,10,11,12,13,14,15,16,17,18,19,20,21,22,23,24,25,26,27]
		Semen/oocytes: determine if there is LSDV in reproductive organs of cattle and bulls, semen, oocytes after experimental infection	6	[28,29,30,31,32,33]
		Direct transmission: detect if there is a direct transmission between experimentally infected animals and healthy animals in a vector-proof environment	2	[34,35]
		Establish the presence of LSDV in meat and offal products	1	[36]
		Establish the spill over from a vaccine	1	[37]
Observational studies				
Cross-sectional studies	Multivariable logistic or regression modelling	Risk factors for LSD outbreaks, i.e., herd size, movement of animals, weather conditions	11	[38,39,40,41,42,43,44,45,46,47,48]
	Ecological niche models Bayesian hierarchical models	Identification of geographic locations and weather conditions which are suitable for the occurrence/spread of LSDV	3	[49,50,51]
	Mathematical modelling	Evaluation of modes of transmission; establish transmission parameters and the R0 between animals	2	[52,53]
	Thin-plate spline regression	Determine the spread rate	1	[54]
	Time series and spectral analysis	Temporal trends and seasonal effects	1	[55]
	Spatial temporal analysis	Evaluate the epidemic between different geographical areas	3	[56,57,58]
	Weather based model	Estimation of population dynamics of potential vectors	1	[59]
	Kernel-based modelling	Determine the force of infection based on distance and seasonality	1	[60]
	Hybrid single particle. Lagrangian-integrated trajectory model	Identify wind events that condition vector transport	1	[61]
Descriptive studies	Field sampling of animals/suspected vectors	Detecting LSDV in animals other than cattle	9	[62,63,64,65,66,67,68,69,70]
		Intrauterine transmission of LSDV in natural conditions	1	[71]
		Semen from naturally infected bulls	1	[72]
		Detection, isolation of vaccine strains	6	[73,74,75,76,77,78]
		Isolation of LSDV in field-collected vector	6	[2,79,80,81,82,83]
Risk Assessment				
Qualitative	WOAH Risk analysis guidelines	Probability of introduction and/or spread into a country considering different pathways	4	[84,85,86,87]
	WOAH Risk analysis guidelines and trade data	Probability of introduction and/or spread into a country considering different pathways	1	[88]
Quantitative	Quantitative import risk analysis	Stochastic model for the probability of LSD introduction in a free country via a specific pathway	2	[89,90]
	Created a generic framework	A single pathway of introduction, i.e., live cattle trade	1	[91]
Literature Review	Literature review	Literature review of the *Stomoxys* fly with additional information of outbreak data	1	[92]

Legend: LSD, Lumpy skin disease; LSDV, Lumpy skin disease virus; PCR; R0, the basic reproduction number; WOAH, World Organization for Animal Health.

**Table 3:** Lumpy skin disease virus detected/isolated per animal species, country and year of sampling.

Animal (Species)	Type of Samples, Test and Location	Country Year of Sampling	Reference
African buffalo (*Syncerus caffer*)	- Sampling of free ranging African buffaloes living close to cattle holdings - 150 out of 254 African buffaloes were seropositive by IFAT to capripox virus - 85 seropositive to LSDV by microserum neutralization test	Kenya 1981	[62]
African buffalo (*Syncerus caffer*)	- Sampling of 248 wild African buffaloes living in a national reserve park - Indirect ELISA test IgG to LSDV detected in 28.2% of samples - Seroneutralization test antibodies to LSDV detected in 7.6% of samples	South Africa 2014	[63]
Egyptian buffalo (*)	- Asymptomatic farmed buffaloes in contact with clinically infected cattle were skin and blood samples - Skin biopsies were tested by real time PCR. Three samples all tested negative - Serum samples were examined using ELISA: 17 out of 96 samples were seropositive.	Egypt 2016 to 2019	[64]
Asian buffalo (*Bubalus bubalis*)	- Clinical examination of LSD suspected cases in buffaloes belonging to small holders. - Detailed findings recorded in a clinical register gave the diagnosis of LSD; two animals were considered positive to LSDV.	India 2020	[65]
Buffalo (*)	- Blood samples collected from buffaloes presenting clinical signs of LSD - 15.2% of blood samples were seropositive to LSDV (type of testing used, e.g., ELISA, seroneutralization not specified)	Egypt 2018	[38]
Buffalo (*)	- Confirm LSD from reported cases in Iraqi buffaloes- PCR: eight positive out of 150 samples - Histopathology of skin lesions of 13 suspected LSD cases: only 1 positive	Iraq 2021 to 2022	[66]
Arabian oryx (*Oryx leucoryx*)	- LSD clinical sign observed in a captive bred female Arabian oryx, at a National Wildlife Research Centre, Saudi Arabia - Neutralizing antibodies used to establish LSD diagnosis in two Oryx (one with -clinical signs and the other without) in a herd of 90 animals. Electron microscopy was used on a single sample from the clinical affected animal. Sample was considered positive	Saudi Arabia 1989	[67]
Southern eland (*Taurotragus oryx*)	- 40 nasal swabs collected from wild ruminants shot during a hunting season on a private farm - Asymptomatic eland tested (two samples only) positive by conventional PCR and real-time PCR for LSDV	Namibia 2019	[68]
Southern eland (*Taurotragus oryx*); Springbok (*Antidorcas marsupialis*); Impala (*Aepyceros melampus*); Wildebeest (*Connachaetes gnou*, *C. taurinus*)	- Serum samples of different free living wild animals in South Africa in the major vegetation zones, i.e., semi-desert, Cape shrub land, grassland, woodland and forest transition - ELISA: serum antibodies detected in 10% of Wildbeest- species *Connachaetes gnou*, 23% of *C taurinus*, 7% of southern eland, 23% of springboks and 20% of impalas	South Africa 1993–1995	[69]
Giraffe (*Giraffa Camelopardalis*)	- Genome detection and isolation of LSDV in a zoo giraffe with LSD clinical signs - Phylogenetic analysis: isolate closely related to the previous Vietnamese and Chinese LSDV cattle strains.	Vietnam 2021	[70]

Legend: (*) article did not specify the buffalo species; DNA = Deoxyribonucleic Acid; IFAT = Indirect Fluorescent Antibody Test; ELISA = Enzyme-Linked Immunosorbent Assay; IgG = immunoglobulin G; LSD = lumpy skin disease; LSDV = lumpy skin disease virus; PCR = polymerase-chain reaction.

**Table 4:** Parameters of insect vectors investigated in the experimental studies and LSDV in field-collected vectors.

Vector Investigated	Detection of LSDV ^(a)^ on the Vector	Detection of LSDV in a Specific Body Part of the Vector	LSD Viral Retention on the Insect	Evidence of LSDV Replication in the Insect	Transmission Attempts of LSDV	Basic Reproduction Number (R0)	Detection of LSDV in Field-Collected Samples
Stable fly							
*Stomoxys calcitrans*	[8,9,10,12,13,14,15]	[10,12,14,15]	[8,9,10,12,13,14,15]	[8,9,10,12,13,14,15]	[8,12,15]	[9,11]	[59,79,80]
*Stomoxys sitiens*	[12,13]	[12]	[12,13]	[12,13]	[12]		
*Stomoxys indica*	[12,13]	[12]	[12,13]	[12,13]	[12]		
Mosquitoes							
*Aedes aegypti*	[9,10,16,17]	[10,17]	[9,10,16,17]	[9,10,16,17]	[16]	[9,11]	
*Anopheles stephensi*	[8]		[8]	[8]	[8]	[11]	
*Culex quinquefasciatus*	[8,9,10]	[10]	[8,9,10]	[8,9]	[8]	[9,11]	
*Culex pipiens*	[17]	[17]	[17]	[17]			
*Aedes japonicus*	[17]	[17]	[17]	[17]			
Biting midges							
*Culicoides nubeculosus*	[8,9,10,17]	[10,17]	[8,10,17]	[8,10,17]	[8]	[9,11]	[39]
*Culicoidess* spp., *C. punctatus*	[17,39]	[17]	[17]	[17]			[17,39]
Horseflies							
*Haematopota* spp.	[15]		[15]		[15]		
*Tabanus bromiums*							[79]
Non biting flies							
*Musca domestica* L.	[81,82]						[81,82]
*Muscina stabulans*	[81]						[81]

Legend: ^(a)^ LSDV = lumpy skin disease virus; R0, the basic reproduction number.

**Table 5:** Type of transmission researched and achieved in tick species in experimental studies.

Type of Infection/Transmission	Tick Species			
	*Amblyoma hebraeum*	*Rhipicephalus appendiculatus*	*Rhipicephalus decoloratus*	*Rhipicephalus annulatus*
Intrastadial infection. Either nymphs or adult ticks without LSDV were allowed to feed on LSD infected cattle and then tested for the presence of the virus (body, or specific organs, e.g., salivary gland, gut)	[20,26,27]	[20,26,27]	[27]	[18] *
Intrastadial/mechanical transmission. Adult ticks are interrupted in their feeding from a cow experimentally infected with LSDV and placed onto susceptible cows which are later tested for LSDV infection (i.e., transmission occurred)	[23]	[25]		
Transstadial persistence. Ticks at the larvae or nymphal stage are fed to repletion on cattle experimentally infected with LSDV. Nymphs then are incubated for molting into adults which are later tested for LSDV presence	[20,21,26,27] **	[20,26,27]		
Transstadial/mechanical transmission. Ticks at the larvae or nymphal stage are fed to repletion in cattle experimentally infected with LSDV. Emerging adult ticks are transferred onto healthy cattle to check if they were infected (i.e., transmission occurred)	[23]	[20]		
Transovarial passage. Female ticks were allowed to feed on LSDV experimentally infected cattle and later incubated to oviposit and for eggs to hatch. Eggs and/or mature larvae were tested for LSDV infection	[22]	[22]	[21,22,27]	[18]
Transovarial transmission. Female adult ticks or larvae were allowed to feed on LSDV infected cattle and later incubated to oviposit and for eggs to hatch. Hatched larvae were place into healthy cows which are later tested to check if they were infected (i.e., transmission occurred)	[22]	[22]	[22,24]	

Legend: * [18] In this study, ticks were collected on naturally infected cattle. ** [21] In this study the LSDV was directly inoculated into the nymphs or adult ticks.

**Table 6:** Risk factors that were identified with LSD occurrence or reoccurrence in the articles retrieved from the systematic literature review.

Identified Main Risk Factors	Country/Region of Study	Reference
*Seasonality*		
Risk of outbreaks increases with higher temperature and/or rainfall	Egypt, Middle East, Balkans, Iran, Ethiopia, Albania, Eurasia, Uganda, Eastern and central Asia, Turkey, Russia	[40,41,42,49,50,51,53,54,55,56,57,60]
*Animal movements or trade*	Egypt, Balkans, Ethiopia, Turkey, Kazakhstan	[39,40,41,43,44,45,54]
*Herd characteristics*		
Type of holdings, i.e., backyard, commercial farms	Turkey, Middle East, Russia	[39,50,56]
Herd size	Ethiopia, Kazakhstan	[43,44]
*Cattle characteristics*		
Age	Mongolia, Egypt, Uganda, Ethiopia, Turkey	[38,40,41,42,46,48]
Breed	Turkey, Egypt, Bangladesh	[38,39,40,47]
Sex	Turkey, Uganda, Mongolia, Bangladesh	[41,42,46,47]
*Farm location/landscape*		
Urban and mixed rain-fed arid livestock system	Middle East	[50]
Areas mostly covered with croplands, grassland or shrub land	Eurasia	[57]
Presence of a water body near the farm (e.g., lake, river, pond, well)	Turkey, Ethiopia, Mongolia	[39,46,48]
Type of agro-climate	Ethiopia	[45,55]
*Type of herd management*		
Water sources: communal or located in farm	Egypt, Uganda, Ethiopia, Mongolia	[42,43,45,46]
Grazing: private or communal/pastoral	Uganda, Egypt, Ethiopia	[40,42,43,45]
Contact of cattle with other animals (e.g., buffaloes, sheep)	Egypt, Uganda, Ethiopia	[40,42,48]
*Cattle density*	Eurasia, Middle East	[50,57]

**Table 7:** Odds ratio retrieved from the studies that used multivariable logistic regression models.

Category Factor	Risk Factor		Odds Ratio (95% C.I.)	Reference
Herd characteristics	Genus/breed	Buffalo Cattle	Reference 4.08 (1.98–8.4)	[38]
		Baladi Mixed Holstein	Reference 4.59 (1.83–11.48) 4.58 (1.73–12.12)	[40]
		Local Cross breed	Reference 3.58 (1.40–9.17)	[47]
	Sex	Male	Reference	
		Female	19.29 (2. 46–151.32)	[41]
			1.72 (1.02–2.92)	[42]
			2.40 (1.11–5.16)	[46]
			3.96 (2.16–7.27)	[47]
	Age	<1 year 1–2 years >2 years	Reference 2.35 (1.48–3.7) 1.33 (0.88–2.01)	[38]
		<1 year 1–3 years >3 years	Reference 1.41 (0.63–3.11) 2.49 (1.17–5.32)	[40]
		>24 months <24 months	Reference 21.1 (8.83–50.43)	[41]
		0–12 months 13–24 months >25 months	Reference 1.24 (0.63–2.44) 1.96 (1.15–3.34)	[42]
		0.5–1 year 1–4 years ≥4 years	Reference 1.38 (0.90- 2.09) 2.44 (1.67- 3.55)	[48]
		Calf Young Adult	Reference 0.21 (0.02–1.71) 0.05 (0.01–0.37)	[46]
	Herd size	Small (2–11 animals) Medium and large (>12 animals)	Reference 19.3 (1.4–50)	[43]
		Small Medium Large	Reference 0.68 (0.54–0.84) 0.63 (0.49–0.81)	[44]
Management	Grazing system	Communal/pastoral Fenced farm Zero grazing	Reference 5.26 (2.64–10.48) 0.28 (0.06–1.44)	[42]
		Separate Communal Both	Reference 1.55 (0.91–2.60) 0.75 (0.39–1.42)	[40]
	Communal water sources	No Yes	Reference 3.28 (2.11–5.09)	[40]
			3.31 (1.42–7.71)	[42]
	Grazing and water sources	Separate/Private Communal	Reference 4.1 (2.02–6.18)	[45]
			14.44 (2.23–94.0)	[43]
	Water source	River Pond Tube well	Reference 0.18 (0.06–0.53) 0.16 (0.05–0.47)	[46]
Management	Free animal movement	No Yes	Reference 0.36 (0.24–0.52)	[40]
	Contact with other animals	No Yes	Reference 3.40 (1.62–7.10)	[40]
			0.41 (0.23- 0.74)	[48]
	Contact with buffalo	Never Daily Weekly/monthly	Reference 1.78 (0.50–6.31) 0.49 (0.29–0.85)	[42]
	New introduction of cattle in the herd	No Yes	Reference 8.5 (6.0–11)	[45]
			2.22 (1.32–3.71)	[40]
			4.43 (2.6–7.5)	[43]
	Purchase of animals	No Yes	Reference 11.67 (8.87–15.35)	[44]
	Sale(s) of animals during LSD outbreaks	No Yes	Reference 1.24 (1.06–1.45)	[44]
	Vaccination	No Yes	Reference 0.13 (0.05–0.34)	[41]
Environment	Season	Winter Autumn Spring Summer	Reference 0.19 (0.02–1.50) 0.87 (0.29–2.51) 7.30 (3.97–13.42)	[40]
	Mean annual rainfall	800–1000 mm 1001–1200 mm 1201–1400 mm	Reference 5.60 (2.35–13.34) 4.58 (2.23–9.40)	[42]

## Data Availability

The data supporting the findings of this study are available from the corresponding authors upon reasonable request.

## Appendix A

PRISMA 2020 Checklist.

**Table:**

Section and Topic	Item #	Checklist Item	Location Where Item Is Reported
TITLE			
Title	1	Identify the report as a systematic review.	1
ABSTRACT			
Abstract	2	See the PRISMA 2020 for Abstracts checklist.	1
INTRODUCTION			
Rationale	3	Describe the rationale for the review in the context of existing knowledge.	1–3
Objectives	4	Provide an explicit statement of the objective(s) or question(s) the review addresses.	3
METHODS			
Eligibility criteria	5	Specify the inclusion and exclusion criteria for the review and how studies were grouped for the syntheses.	3–4
Information sources	6	Specify all databases, registers, websites, organizations, reference lists and other sources searched or consulted to identify studies. Specify the date when each source was last searched or consulted.	3–4
Search strategy	7	Present the full search strategies for all databases, registers and websites, including any filters and limits used.	4
Selection process	8	Specify the methods used to decide whether a study met the inclusion criteria of the review, including how many reviewers screened each record and each report retrieved, whether they worked independently, and if applicable, details of automation tools used in the process.	3 (Table 1)
Data collection process	9	Specify the methods used to collect data from reports, including how many reviewers collected data from each report, whether they worked independently, any processes for obtaining or confirming data from study investigators, and if applicable, details of automation tools used in the process.	3
Data items	10a	List and define all outcomes for which data were sought. Specify whether all results that were compatible with each outcome domain in each study were sought (e.g., for all measures, time points, analyses), and if not, the methods used to decide which results to collect.	-
	10b	List and define all other variables for which data were sought (e.g., participant and intervention characteristics, funding sources). Describe any assumptions made about any missing or unclear information.	-
Study risk of bias assessment	11	Specify the methods used to assess risk of bias in the included studies, including details of the tool(s) used, how many reviewers assessed each study and whether they worked independently, and if applicable, details of automation tools used in the process.	3
Effect measures	12	Specify for each outcome the effect measure(s) (e.g., risk ratio, mean difference) used in the synthesis or presentation of results.	Not appropriate
Synthesis methods	13a	Describe the processes used to decide which studies were eligible for each synthesis (e.g., tabulating the study intervention characteristics and comparing against the planned groups for each synthesis (item #5)).	-
	13b	Describe any methods required to prepare the data for presentation or synthesis, such as handling of missing summary statistics, or data conversions.	-
	13c	Describe any methods used to tabulate or visually display results of individual studies and syntheses.	-
	13d	Describe any methods used to synthesize results and provide a rationale for the choice(s). If meta-analysis was performed, describe the model(s), method(s) to identify the presence and extent of statistical heterogeneity, and software package(s) used.	-
	13e	Describe any methods used to explore possible causes of heterogeneity among study results (e.g., subgroup analysis, meta-regression).	-
	13f	Describe any sensitivity analyses conducted to assess robustness of the synthesized results.	Not appropriate
Reporting bias assessment	14	Describe any methods used to assess risk of bias due to missing results in a synthesis (arising from reporting biases).	Not appropriate
Certainty assessment	15	Describe any methods used to assess certainty (or confidence) in the body of evidence for an outcome.	Not appropriate
RESULTS			
Study selection	16a	Describe the results of the search and selection process, from the number of records identified in the search to the number of studies included in the review, ideally using a flow diagram.	4.5
	16b	Cite studies that might appear to meet the inclusion criteria, but which were excluded, and explain why they were excluded.	4
Study characteristics	17	Cite each included study and present its characteristics.	6 Appendix B
Risk of bias in studies	18	Present assessments of risk of bias for each included study.	Appendix B
Results of individual studies	19	For all outcomes, present, for each study: (a) summary statistics for each group (where appropriate) and (b) an effect estimate and its precision (e.g., confidence/credible interval), ideally using structured tables or plots.	6–20 Appendix B
Results of syntheses	20a	For each synthesis, briefly summarize the characteristics and risk of bias among contributing studies.	6–20
	20b	Present results of all statistical syntheses conducted. If meta-analysis was performed, present for each the summary estimate and its precision (e.g., confidence/credible interval) and measures of statistical heterogeneity. If comparing groups, describe the direction of the effect.	6–20 No meta-analysis
	20c	Present results of all investigations of possible causes of heterogeneity among study results.	6–20
	20d	Present results of all sensitivity analyses conducted to assess the robustness of the synthesized results.	Not appropriate
Reporting biases	21	Present assessments of risk of bias due to missing results (arising from reporting biases) for each synthesis assessed.	Not appropriate
Certainty of evidence	22	Present assessments of certainty (or confidence) in the body of evidence for each outcome assessed.	6–20
DISCUSSION			
Discussion	23a	Provide a general interpretation of the results in the context of other evidence.	6–20
	23b	Discuss any limitations of the evidence included in the review.	6–20
	23c	Discuss any limitations of the review processes used.	6–20
	23d	Discuss implications of the results for practice, policy, and future research.	21–26
OTHER INFORMATION			
Registration and protocol	24a	Provide registration information for the review, including register name and registration number, or state that the review was not registered.	Not registered
	24b	Indicate where the review protocol can be accessed, or state that a protocol was not prepared.	-
	24c	Describe and explain any amendments to information provided at registration or in the protocol.	-
Support	25	Describe sources of financial or non-financial support for the review, and the role of the funders or sponsors in the review.	26
Competing interests	26	Declare any competing interests of review authors.	26
Availability of data, code and other materials	27	Report which of the following are publicly available and where they can be found: template data collection forms; data extracted from included studies; data used for all analyses; analytic code; any other materials used in the review.	Appendix B

## Appendix B

List of the publications included in the systematic review, with description of study type, purpose main methodology, main findings and conclusions and limita-tions of the study, and geographical area of where the study was carried.

**Table:**

Ref.	Author/Year	Type of Study	Study Purpose	Methodology	Main Findings/Conclusions	Limitations of the Study	Geographical Area of Study
[2]	Yeruham et al. (1995)	ObD Vec-Ins	To describe the conditions and dairy herds affected by LSD ^(a)^ outbreaks.	Description of the area and herds in which LSD outbreaks were reported. Haematology, biochemistry and serology were performed on blood samples collected from affected animals, along with histopathology of skin lesions. Local wild ruminants, i.e., gazelles (*Gazella gazella*), and sheep and goats were examined in search of LSD clinical signs.	It concluded that although the origin of the LSD outbreak in the dairy herds could not be traced with certainty, the circumstantial evidence (no cattle newly introduced in the village herds, thus, other means of introduction were therefore suggested) indicated that the LSDV ^b^ was brought from Egypt by wind-carried *Stomoxys calcitrans*.	Results are not very detailed. The study only mentions the number of herds affected but not the number of cattle heads. The study only describes the epidemiology of the first LSD outbreak in Israel. Thus, all inferences on the modes of transmission and spread of the disease in the dairy herds were conducted using circumstantial evidence.	Israel
[62]	Davies (1982)	ObD Host	Attempts to define the maintenance of LSD in hosts living in high altitude indigenous forests by searching for antibodies to LSD virus in the sera from wild and domestic ruminants.	Blood samples of cattle and wild ruminants were collected from different sources for LSDV isolation and serology through microserum neutralization tests and indirect fluorescent antibody test.	The African buffalo (*Syncerus caffer*) had Abs to capripox virus: out of 254 buffaloes, 150 animals were seropositive to IFAT, along with a small number of domestic cattle. An LSD endemic area was proposed and authors suggested that the maintenance cycle involves the buffalo. No Ab was detected in the other wild ruminant species investigated. It concluded that, while an epidemic of LSD has occurred in Kenya, most cases were sporadic and probably the result of accidental contacts with a component of the maintenance cycle.	Serology testing cannot distinguish the three viruses in the Capripoxvirus genus (sheep pox virus, goat pox virus and LSDV). The period of study and geographical environment were described. In the results, authors indicated that from the sera from positive to the IFAT test (150 out of 254) three groups of buffalo sera contained a significant number which neutralized the LSD/2490 strain of virus. There was no neutralization of cowpox virus by any of these positive sera, which increases the likelihood that the neutralization of LSD is due to specific antibody and not due to non-specific neutralizing properties of the sera. This is not a confirmation and the number of buffaloes was not specified.	Kenya
[63]	Fagbo et al. (2014)	ObD Host	To expand the understanding of the role of buffalo in the maintenance of LSDV and Rift Valley Fever (RVF) by determining their seroprevalence during an inter-epidemic period.	Between 2003 and 2004, blood samples were collected from African buffaloes in the Kruger National Park and Hluhluwe-iMfolozi Park, South Africa. They were tested for IgG Abs for LSD with ELISA ^(c)^ and positive or suspected positive samples were further tested by SNT ^(d)^	The I-ELISA for LSDV and RVFV ^(e)^ detected IgG antibodies in 70 out of 248 (28.2%) and 15 out of 248 (6.1%) buffaloes, respectively. Using the SNT, LSDV and RVFV neutralizing Abs were found in 5 out of 66 (7.6%) and 12 out of 57 (21.1%) samples tested, respectively. Authors suggested that African buffaloes play a role in the epidemiology of these diseases during inter-epidemic periods.	Limitations with serological tests as it is not possible to distinguish the three viruses in the Capripox virus (sheep and goat pox viruses and LSD). The SNT, only gave 5 positive out of 66 samples, i.e., the gold standard did not compare correctly with results obtained by the I-ELISA used in the study, as the I-ELISA is not validated for wildlife sera. Authors mention that the African buffalo plays a role in the inter-epidemic period but the specific sampling period of the year (e.g., during the rainy or dry season, during an outbreak in the country) was never specified, thus it was not possible to draw that conclusion.	South Africa
[64]	Ahmed et al. (2021)	ObD Host	To identify and characterize the LSD virus outbreaks in Egypt, between 2016 and 2019, and determine the role of Egyptian buffaloes in the epidemiology of LSD.	Forty-one and three skin biopsies were performed on clinically-affected cattle and buffaloes, respectively; 31 blood samples were collected from asymptomatic buffaloes in contact with clinically-infected cattle and tested by RT-PCR ^(f)^. Samples were collected from 102 bovines showing clinical signs of LSD and 96 Egyptian buffaloes, with no vaccination history, and in contact with LSD clinically-affected cattle. Positive samples were isolated and sequenced; phylogenetic trees were constructed.	Among the skin biopsies that underwent RT-PCR to detect LSDV, 31 cattle heads were positive and all buffaloes were negative. LSDV was isolated on CAM and MDBK cell culture in 19 positive samples. ELISA results: 84/102 cattle were positive and 17/96 buffalo were positive. The phylogenetic analysis was identical for all isolates, and presented a 99–100% identity with LSDV isolates from different countries in Africa, Asia, and Europe. ELISA analyses detected sero-reactivity to LSDV in Egyptian cattle and buffaloes. Conclusion: the Egyptian water buffalo is an accidental, non-adapted, host of the virus and the current vaccine strategy for LSD control should be re-evaluated to improve coverage and effectiveness.	Although it is proposed that Egyptian buffaloes are less susceptible to LSDV infection, only 3 samples of skin biopsies were used to confirm the presence of LSDV by RT-PCR. Antibodies were also detected by ELISA, but a low percentage were positive. These differences in results could be explained by a number of factors (e.g., sensitivity, specificity of the ELISA, Ab’s were produced due to another Capripoxvirus, low number of skin biopsies tested) which are not elaborated in the article.	Egypt
[65]	Pandey et al. (2022)	ObD Host	To highlight the speed at which the disease can spread in animal populations, previously presumed to be naïve, and to quantify its impact with reference to subsistence agriculture in rural communities.	Clinical signs were described and recorded after full clinical examination of affected animals (oxen, cows, *Bos indicus* calves and Asian water buffalo), in small village holdings around a tiger reserve. Questionnaires allowed gathering information on the clinical disease history and animal husbandry practices relevant to the spread of LSDV.	The signs of LSD were recorded and described in 154 oxen, 34 cows, 13 calves (*Bos indicus*) and two Asian water buffaloes (*Bubalus bubalis*). The description of an LSD outbreak in naïve populations of cattle and buffaloes illustrated the need for increased awareness on the associated clinical signs and the maintenance of high biosecurity levels in hitherto disease-free countries.	Diagnosis of LSD only relied on clinical signs, which could lead to false positives or negatives and thus to an over- or underestimation of the prevalence.	India
[38]	Faris et al. (2021)	ObC RiskF.	To assess the prevalence of LSD in five selected localities in an Egyptian governorate and to detect the potential risk factors associated with LSD.	Blood samples were collected from 599 cattle heads and 66 buffaloes, with and without clinical signs of LSD. Temperature humidity index (THI), resulting from the combination of air temperature and humidity, associated with the level of thermal stress was calculated. A multivariate logistic regression assessed the risk factors related to LSD prevalence. The risk factors identified in the study were: animal species (cattle and buffaloes), age, season (winter, spring and summer), THI, locality and immune status of animals (vaccinated vs. unvaccinated).	The prevalence was 36.7% in cattle and 15.2% in buffaloes. Regarding the influence of age, the prevalence was 26.3% in animals <1 year, 42.2% in animals aged 1–2 years and 34.9% in the >2 years group. When considering the season, the prevalence reached 29.3% in the winter, 34.1% in the spring and 37.7% in the summer. A prevalence of 29.7%, 31.6% and 37.6% were calculated for a low, moderate and high THI, respectively. The prevalence in vaccinated vs. unvaccinated animals was 34.3% vs. 50%. The authors concluded that LSD had become endemic in Egypt and was responsible for sporadic outbreaks over the year, mainly in adult animals and during the summer; cattle was more likely to be infected than buffalo.	The study assessed LSD prevalence in five localities using blood samples but no information on the diagnostic test used to confirm an LSDV infection was provided. Additionally, the authors did not specify if farmed cattle was randomly. Furthermore, no sample size was calculated. The season explanatory variables group did not include autumn and no explanation was given for its exclusion.	Egypt
[66]	Aboud et al. (2022)	ObD Host	To confirm infection of Iraqi buffaloes with LSDV.	Blood samples, clinical examination to detect skin lesions and collection of ticks from 150 buffaloes of different ages and sexes. Tests used: PCR ^(g)^ and histopathology.	Eight out of 150 buffaloes were positive by PCR. The histopathology performed on skin lesions revealed that one out of 13 samples were positive to LSDV. Among 29 ticks (species not specified) collected, none was positive. This is the first study to investigate LSD in buffaloes, to identify positive animals and to describe rare clinical signs. It concluded that an effective control of LSD requires an accurate and rapid laboratory diagnostic method such as PCR; histopathology could be a method to identify and confirm the disease along with clinical examination.	Sampling was not random. Animals were selected based on information provided by veterinarians and buffalo owners who observed the clinical signs. Only 13 LSD-suspect skin were sampled and analyzed via histopathology. The tick sample was very small (N = 29) and authors did not explain why.	Iraq
[67]	Greth et al. (1992)	ObD Host	Sampling of captive-bred Arabian Oryx (*Oryx leucoryx*) from a national wildlife research center after an animal showed clinical signs of LSD.	Serology survey; virus was identified by electron microscopy. Virus neutralization was performed by antibody titer on paired sera.	It was the first case of LSD infection described in the Arabian oryx, and also the first case reported in Saudi Arabia. The serologic survey of the herd (90 oryx) showed a low prevalence (2%) of infection and only one out of the two positive animals developed lesions.	Sampling was performed in captive animals (i.e., not living freely), so the role of wildlife cannot be ascertained. The presence of LSDV was not confirmed by the tests used. The only certainty was that a *Capripox* virus was involved.	Saudi Arabia
[68]	Molini et al. (2021)	ObD Host	To assess the presence of LSD in Namibian wildlife, the disease being endemic in cattle in the area.	Nasal swabs and DNA samples, tested by PCR and RT-PCR, were collected from wild ruminants shot during the hunting season on a private farm in Namibia.	Only one sample from an asymptomatic eland (*Taurotragus oryx*) tested positive, out of 12 different wild animals. This is the first evidence of the presence of LSDV DNA in an eland. Forty swabs were analyzed, two were from eland.	Although there was a limitation on the number of sampled animals, this confirmed a case of LSDV in a wild animal. No clinical signs were observed so the status of wild animals as reservoirs of LSD remains to be further investigated.	Namibia
[69]	Barnard (1997)	ObD Host	To investigate the possibility that game animals (i.e., animals raised for hunting) are involved in the epidemiology of some of the most common viral diseases of livestock In South Africa.	Authors tested 24 species of South African wild animals for the presence of Abs against 16 common viruses of domestic animals, including LSDV. Standard serological tests were used. The average annual rainfall of the sampling area was calculated, over a 20 year-period.	The results of LSD prevalence, based on ELISA testing, were the following: 10% in black wildebeests (3/31 positive), 27% in blue wildebeests (4/15 positive), 23% in springboks (12/53 positive), 20% in impalas (5/25) and 7% in elands (1/15). The prevalence in the different zones varied from 17% in the grassland to 33% in the forest transition area.	There is a limitation in using an LSDV serological test, i.e., it cannot confirm if Abs are synthetized vs. LSDV or vs. goat poxvirus. The test results are shown as positive or negative, but the cut-off was not properly defined (authors refer to their many years of experience with the test used for domestic animals). This could generate true or false positive or negative samples. Only 15 buffalo samples were tested and they were all negative. This sample size was not representative of the real population of buffaloes potentially infected in the national park.	South Africa
[70]	Dao et al. (2022)	ObD Host	To investigate the cause of death of a giraffe in a zoo.	Swab samples were collected from skin nodule biopsies and ruptured nodule wound for LSDV isolation.	It is the firstly reported detection and isolation of LSDV genome in a sick giraffe. The phylogenetic analysis of the isolate showed its close relationship with previous Vietnamese and Chinese LSDV cattle strains.	The source of infection of the giraffe was unknown; the authors presumed contacts with infected cattle but never confirmed such hypothesis.	Vietnam
[34]	Carn and Kitching (1995)	Exp. R.T.	To attempt a transmission of LSDV from infected to susceptible cattle housed in close contact, in order to establish the potential for LSDV to spread in the absence of arthropods.	Cattle was inoculated by three routes, consistent with a mechanical arthropod-borne transmission: on the conjunctival sac, intra-dermally and intravenously. Seven non-infected animals were housed in contact with infected animals for one month, in an insect-proof facility. Virus neutralization tests were performed to confirm the infection. Different contact experiments were carried through.	No susceptible animal became positive. The conclusion was that the transmission of LSDV between animals by direct contact is extremely inefficient, and that a parenteral inoculation of the virus is required. The high proportion of animals who developed a generalized disease after intravenous inoculation implied that field cases of generalized LSD may follow a spread by blood-feeding arthropods.	The study relied on an experimental infection, thus cattle are inoculated with a virulent strain and at high titers. The number of animals used in the experiment was low and the length of the contact period may not have been sufficient.	Not applicable
[52]	Magori-Cohen et al. (2012)	ObC R.T	To evaluate LSD transmission via direct and indirect contact in field conditions.	Using mathematical tools, transmissions via direct and indirect contact in field conditions were compared. A transmission model assessed outbreak dynamics and risk factors for LSD. Data were collected during the 2006-LSD outbreak reported in a large Israeli dairy herd, which included ten separated cattle groups. Transmission by three contact modes was modelled, i.e., indirect contacts between the groups within a same herd, direct contacts or contacts via common drinking water within the groups, and transmission by contact during milking.	Indirect transmission was the only parameter that could solely explain the entire outbreak dynamics; its estimated overall effect was >5 times larger than all other combined routes of transmission. A 15.7-R0 ^(h)^ (basic reproduction number) was induced by the indirect transmission from an infected cow remaining for one day in the herd, while the R0 induced by direct transmission was 0.36. These results indicated that LSDV spread within the herd could hardly be attributed to direct contacts between cattle or contacts during milking. The authors therefore concluded that transmission mostly occurs by indirect contact, probably by flying blood-feeding insects. This conclusion has important implications for the control of LSD.	The epidemic in Israel was swiftly controlled. Hence, clinically affected animals were removed promptly and the herd was vaccinated, which may have affected the transmission parameters.	Israel
[35]	Aleksandr et al. (2020)	Exp. R.T.	To assess the transmission by direct contact among infected and non-infected cows, in an insect-proof facility.	This 60-day experiment involved five inoculated bulls (‘IN’ group) and two groups of in-contact animals (five cows per group, named C1 and C2). Cows belonging to C1 were in contact with the inoculated animals at the onset of the trial while C2 cows were introduced at day 33 of the experiment. The bulls were aged 6–8 months and were inoculated with the virulent vaccine-derived recombinant LSDV strain (Saratov/2017).	The infection in both groups of contact animals was confirmed clinically, serologically and virologically. Viremia was demonstrated in blood, nasal and ocular excretions, using molecular tools. This is the first evidence of an indirect transmission for a naturally occurring recombinant LSDV isolated from the field. Further studies on LSDV biology are a priority: it is important to gain insights on whether the hypothesized indirect contact evidenced in this study is a de novo-created feature, absent from both parental strains of the novel (recombinant) LSDV isolate, or whether it was dormant but unlocked by genetic recombination.	The virulent vaccine-derived recombinant LSDV strain (Saratov/2017) was directly inoculated to the experimental animals. As in other experimental studies, it is hard to establish conditions similar to the field. The virulent character of the strain may have helped the direct transmission.	Not applicable
[28]	Osuagwuh et al. (2007)	Exp S.T.	To determine whether the LSD vaccine strain is excreted in semen after vaccination with modified live vaccines, and to determine the efficacy of vaccination in preventing LSDV excretion in semen of experimentally infected vaccinated bulls.	Six unvaccinated and six vaccinated bulls were infected 27 days after the second vaccination with an LSD modified live vaccine. Furthermore, six unvaccinated bulls were infected experimentally with a virulent LSDV field strain. Blood and semen samples from the bulls were tested by serum neutralization test, virus isolation and PCR.	Vaccinated bulls infected in laboratory conditions tested negative, while unvaccinated bulls were infected. Viral nucleic acid was detected in the severely affected bulls from day 10 post-infection (p.i.) ^(i)^ until 28 p.i., end of the trial. LSDV was detected in semen of unvaccinated infected bulls, thus, the vaccine protect against the spread of LSDV via semen.	A virulent strain was used and semen was tested when clinical signs of LSD were present. Although it is interesting to give insights on the seminal transmission, the field situation is unknown, i.e., the amount of LSDV recovered in semen of naturally infected bulls and the excretion dynamics are unknown.	
[29]	Irons et al. (2005)	Exp S.T.	To establish the incidence and duration of LSDV excretion in the semen of naive bulls infected experimentally.	Semen samples from six bulls experimentally infected with a virulent field isolate were collected intermittently over a 90-day period. Semen was collected for testing until three consecutive samples were found to be negative for LSDV by PCR or until the end of the testing period. Authors conducted virus isolation and tested the infectivity of semen titration in tissue cultures.	All semen samples were LSDV-positive by PCR. The virus was only isolated in two severely affected bulls. This study confirmed the excretion of LSDV in bovine semen for prolonged periods (up to 159 days p.i.) even when obvious clinical signs of the disease were no longer apparent.	The experimental infection used a virulent field isolate. Only six bulls were used. Although all samples were PCR-positive, the virus was only isolated from two severely affected bulls. Although it was isolated by PCR over an extended period, it is unknown how infective the virus is in semen. Indeed, titration to determine the infectivity of viral particles was performed in tissue cultures of a single positive sample, i.e., a bull with obvious clinical signs.	
[72]	Sudhakar et al. (2020)	ObD S.T.	Authors reported the first occurrence of LSD in cattle in India; they analyzed the epidemiological and genetic characterization data from LSD outbreaks in the districts of an Indian state.	Clinical data were collected in the field. Sampling (blood, scab), was performed on 60 cattle showing clinical signs of LSD. Seventeen samples of frozen bull semen were obtained from a semen bank farm. DNA extraction, conventional and real-time PCR and phylogenetic analysis were performed.	The study established the presence of LSDV in India the and involvement of LSDV field strains in the outbreaks. It provided evidence of LSDV shedding in semen of naturally infected bulls; 20.45% of frozen bull semen samples were positive.	This is a descriptive study, thus only circumstantial inferences can be established. The provenance of the frozen field samples was not explained (i.e., small holdings, type of insemination, natural vs. artificial or mixed, dairy or beef herds), thus the effectiveness of seminal transmission under natural conditions has yet to be established.	India
[30]	Annandale et al. (2010)	Exp S.T.	To determine the site of persistence of LSDV in bulls shedding the virus in semen for more than 28 days; to determine if the virus is present in all semen fractions and to study the lesions that develop in the genital tract.	Six bulls were infected. Bulls that were PCR-positive on the whole semen sample collected on day 28 p.i. were slaughtered; tissue samples from their genital tracts were submitted to histopathology, electron microscopy, immune-peroxidase staining, virus isolation and PCR.	Viral DNA was identified in all semen fractions from all bulls, but mostly from the cell-rich fraction and from the severely affected bulls. The PCR assay was positive on post-mortem samples of testes and epididymides 28 days p.i., from the two severely affected bulls. The authors isolated the virus from the testes of both bulls and from the epididymis of one of them. This study suggests that the testis and epididymis are sites of viral persistence in bulls shedding LSDV in semen for prolonged periods and revealed that viral DNA is present in all fractions of the ejaculate.	The time of animal slaughtering conditioned the experimental infection. How long the virus remains in testes and epididymides still needs to be determined, as well as the way it would affect seminal transmission to a heifer.	
[31]	Annandale et al. (2014)	Exp S.T.	Whether LSDV transmitted through semen can infect cows and their embryos.	The authors performed two controlled trials simultaneously. Eleven beef heifers were synchronized and inseminated with fresh semen spiked with LSDV strain on day 0. Six animals were super-ovulated on day 1, then embryos were flushed from these heifers on day 6. Blood and serum samples were collected from day 4 until day 27 to determine the presence of LSDV and Abs. LSDV was detected by PCR, virus isolation or electron microscopy in blood, embryos and in the organs of experimentally infected animals.	LSD was detected in blood, embryos and organs of experimentally infected heifers. This is the first report of experimental seminal transmission of LSDV in heifers and embryos through artificial insemination, thereby confirming the biological risk posed by LSDV-infected semen.	The first positive SNT samples was detected 9 days p.i., whilst Irons et al., 2005 detected by 12 days p.i. This illustrates the variability in experimental studies, such as using a higher viral load in this study, or intra-uterine route of infection (previous was intravenously), which allows different exposures to the immune system.	
[32]	Annandale et al. (2019)	Exp S.T.	To examine the effects of LSDV in frozen-thawed semen on in vitro embryo production parameters, including viral status of media and resulting embryos.	Bovine oocytes were harvested from abattoir-collected ovaries and split into three experimental groups. After maturation, the oocytes were fertilized in vitro with frozen-thawed semen spiked with a high (HD) or a low (LD) dose of LSDV, or with LSDV-free semen (control). Eight day-blastocysts were examined for LSDV by PCR and virus isolation.	The presence of LSDV in frozen-thawed semen reduced embryo yield significantly. Moreover, the presence of the virus in 8-day blastocysts confirmed that embryo transfer is a potential risk of virus transmission in cattle.	Semen was infected in the laboratory, thus frozen immediately post infection after different dosages of LSDV. Although it clearly shows that frozen bull semen could be a mode of transmission, the risk of generating an LSD outbreak should be assessed. Embryos tested positive only by day 8, thus what happens after implantation and how viable this route of transmission is are still unknown. The laboratory conditions could confound a lower yield, and the optimal conditions to obtain viable embryos are not specified.	Not applicable
[33]	Annandale et al. (2018)	Exp S.T.	To investigate the ability of common semen processing techniques to remove LSDV from cryopreserved bull semen, and to investigate the way the virus associates with the sperm cell.	A semen sample was collected from an LSDV-negative bull and divided in three parts, two of which were spiked with different LSDV concentrations, i.e., large and small dose, and third one used as control. Samples were cryopreserved and later unfrozen using different processing methods (swim-up, single-layer centrifugation, Percoll gradient and Percoll gradient with trypsin). Semen evaluation methods for motility, PCR analysis, isolation and electron microscopy were performed on the unfrozen sperm.	None of the common semen processing methods tested were able to clear (i.e., not effective) spiked frozen-thawed bull semen with LSDV, except for the Percoll gradient with added trypsin, but the semen quality was significantly deteriorated. That poses a biosecurity issue in the semen trade. It is unknown whether the concentrations of LSDV used in the study are comparable to those found in bulls naturally infected and shedding the virus in their semen.	Authors used laboratory infected semen, thus frozen immediately after infection by a virulent strain at different concentrations. Although it clearly shows that frozen bull semen could be a mode of transmission, it should further be tested for cow insemination, in order to determine if there is a risk of introducing LSD into a free area via highly contaminated semen.	Not applicable
[71]	Rouby and Aboulsoud (2016)	ObD I.U.	To describe the clinical, histopathological, molecular and serological diagnostic of LSD in a premature one day old-calf, delivered from a cow with clinical signs of LSD.	Description of the clinical, histopathological, molecular and serological diagnosis of LSD in the calf. PCR and gene sequencing confirmed the ELISA and serum neutralization tests.	SNT confirmed that the one day old-calf had developed pre-colostrum serum Abs to LSDV, which indicated virus transmission in utero. All sera collected from animals located in the same area were serologically positive, which confirmed an exposure to LSDV.	Description of a single case study. Although the authors inferred that LSD transmission occurred in utero, they did not explain why only one single calf exhibited clinical signs in the whole herd. It is unknown at what stage of pregnancy the cow was infected. This transmission route is viable but may be affected by other conditions.	
[36]	Kononov et al. (2019)	Exp Meat and offal	To determine the potential presence of infectious virus and genetic material in meat and offal products, including testicles, from sub-clinically and clinically ill cattle inoculated with a virulent LSDV strain.	Fourteen 6 to 7 month-old bulls were infected with LSDV. Infected animals were culled at 21 days p.i. and samples were collected from muscles, skin nodules, lymph nodes, tongue, trachea, lungs, heart, parenchymal organs, rumen, reticulum, omasum, small and large intestine and testicles. Real time-PCR was performed on the samples to detect LSDV.	Findings demonstrated that lymph nodes and testicles of clinically and sub-clinically infected animals are reservoirs of live LSDV, whereas deep skeletal meat in both types of infection does not harbor live virus; the risk of transmission through this product is thus probably very low. The detection of LSDV in testicular tissues in sub-clinically ill animals is a concern, because of the potential spread of the virus through contaminated semen.	Experimental infections used a virulent strain. The bulls were culled at an age different from the usual culling age for meat. Nevertheless, the study showed that the risk of virus transmission via sub-products was low.	
[8]	Chihota et al. (2003)	Exp. Vec.I.	To investigate the transmission of LSDV from infected to susceptible animals by two species of mosquitoes, the stable fly and a species of biting midge.	The mosquitoes *Anopheles stephensi* and *Culex quinquefasciatus*, the stable fly *Stomoxys calcitrans* and the biting midge *Culicoides nubeculosus* were allowed to feed on either LSD-infected animals or through a membrane on a blood meal containing LSDV. These arthropods were then allowed to feed on susceptible cattle at various intervals after the infective meal. Virus was searched for in the insects by PCR.	The LSDV was not transmitted from infected to susceptible animals by *An. Stephensi*, *S. calcitrans*, *C. nubeculosus* and *Cx. Quinquefasciatus*. The transmission was attempted 24 h post-feeding. Inferences were that *S. calcitrans* may act as a mechanical vector of LSDV through interrupted feeding over 1–12 h periods, and not over longer periods. In *C. nubeculosus* midges, LSDV was not detected beyond day 0 post-feeding; the latter was not able to act as a biological vector as there was no evidence of virus replication. Mosquitoes may need to feed on a viraemic lesion to allow transmission. Authors suggested a far more elegant mode of transmission than a mere “dirty-pin” type of virus transfer. Overall, the insect species assessed in the study may be able to transmit LSDV to susceptible animals if their meal on an infected host is interrupted and they have to complete it on another susceptible animal, which is consistent with a mechanical transmission.	Vectorial capacity and competency can be overestimated in experimental studies, i.e., animals are experimentally infected with a virulent strain, hence, have a higher viral load. Furthermore, the experimental hosts are shaved and put into adequate dispositions. Vectors feed when the animals show clinical signs, and at determined points of viremia, they are fed directly with infected blood or directly in a shaved portion of the animal skin or lesion. All these factors artificially increase the capacity and competence of vectors.	Not applicable
[9]	Sanz-Bernardo et al. (2021)	Exp. Vec.I.	Authors used a highly relevant experimental LSD infection model, in the natural cattle host, and four representative blood-feeding insect species previously reported to have the capacity of acquiring LSDV. The study aimed at assessing their acquisition and retention of LSDV, and determining the LSDV R0 in cattle for each model insect species.	Eight cattle were infected by intravenous and intradermal inoculation and all were exposed to: two mosquito species, i.e., *Ae. Aegypti* and *Cx. Quinquefasciatus*, *C. nubeculosus* biting midge and to the stable fly *S. calcitrans* on different days. Based on these quantitative data, and by combination with data from other studies, the authors used mathematical models to determine the R0 of LSDV in cattle, as mediated by each of these insect species.	The probability of vectors acquiring LSDV from a sub-clinically-infected animal was very low (0.006) compared with an animal showing clinical signs (0.23). It means an insect feeding on a sub-clinically-infected animal was 97% less likely to acquire LSDV than one feeding on an animal showing clinical signs. These four potential vector species acquired LSDV from the host at a similar rate, but *Ae. Aegypti* and *S. calcitrans* retained the virus for a longer time, i.e., up to 8 days. There was no evidence of virus replication in the vectors, which is consistent with a mechanical rather than a biological transmission. The R0 was highest for *Stomoxys calcitrans* (19.1), followed by *C. nubeculosus* (7.1) and *Ae. Aegypti* (2.4), indicating that these three species are potentially efficient vectors of LSDV.	Same limitations as experimental study [8].	Not applicable
[10]	Sanz-Bernardo et al. (2022)	Exp. Vec.I.	To add results from a previous study on the role of hematophagous insects in the transmission of LSDV. The authors investigated the vector-borne transmission of LSDV in more details, by quantifying the acquisition and retention of LSDV in different anatomical parts of four vector species.	Four vector species were focused on: *S. calcitrans*, *Ae. Aegypti*, *Cx. Quinquefasciatus* and *C. nubeculosus.* They were fed on either a lesion, normal skin of experimentally infected- cows or on an artificial membrane system containing viraemic blood. After feeding, insects were incubated for 0, 2, 4, or 8 days and then dissected into proboscis, head-thorax (including the upper digestive tract and salivary glands), and abdomen or proboscis and head-thorax-abdomen. The DNA of LSDV was searched for by PCR; LSDV titration was performed in skin biopsy. Mathematical models were generated to establish the parameters that influence the acquisition and retention of LSDV, by insects.	For the four insect species, the probability of acquiring LSDV was substantially greater when feeding on a lesion compared with feeding on normal skin or blood from an animal showing clinical signs. After feeding on a skin lesion, LSDV was retained on the proboscis for a similar length of time (around 9 days) for the four species and for a shorter time in the rest of the body, ranging from 2.2 to 6.4 days. The insect body, rather than the proboscis, was more likely to be positive immediately after feeding. Acquisition and retention of LSDV by *Ae. Aegypti* after feeding on an artificial membrane feeding system that contained a high titre of LSDV was comparable to feeding on a skin lesion on an animal showing clinical signs, supporting the use of this laboratory model as a replacement in some animal studies. The probability of acquiring LSDV was highest for *S. calcitrans*, followed by *Ae*. *Aegypti*, *Cx*. *Quinquefasciatus* and *C. nubeculosus*.	Same limitations as experimental study [8].	Not applicable
[11]	Gubbins et al. (2019)	Exp. Vec.I.	To estimate the risk of LSDV transmission by five different species of biting insects, based on the R0.	The R0’s related to the mechanical transmission of LSDV were estimated based on previously published data of transmission experiments. Vector life history parameters were derived from published literature. The five species of biting insects were: the stable fly *S. calcitrans*, the biting midge *C. nubeculosus*, and three mosquito species, i.e., *Ae. Aegypti*, *An. Stephensi*, and *Cx. Quinquefasciatus*.	With regard to R0 median (95% confidence interval), the results of skin lesions were the following: *S*. *calcitrans* 15.5 (1.4–81.9), *Ae. Aegypti* 7.4 (1.3–17.6), *C*. *nubeculosus* 1.8 (0.06–13.5), *An. Stephensi*, 1.6 (0.2–6.0) and *Cx*. *Quinquefasciatus* 0.8 (0.09–3.5). The results suggest that *S. calcitrans* is likely to be the most efficient in transmitting LSDV, but *Ae*. *Aegypti* would also be an efficient vector. By contrast, *C*. *nubeculosus*, *An*. *Stephensi* and *Cx*. *Quinquefasciatus* are likely inefficient vectors of LSDV.	These parameters were estimated based on literature data, in particular, from experiments focusing on LSDV transmission by the five putative vector species. Parameters from the literature could vary as vector competence studies provided variable results.	Not applicable
[59]	Kahana-Sutin et al. (2017)	ObC Vec.I.	To assess the possible vector(s) of LSDV under field conditions.	A year-round trapping of dipterans was implemented in 12 Israeli dairy farms, one year after LSD outbreaks. Their abundance was compared with their abundance at the onset of 2012- and 2013-outbreaks, under the assumption that vector seasonality remains approximately the same over the years. Vector and environmental data were added to a weather-based model to explain the trapping results.	The relative abundance of *S. calcitrans* during the outbreak period (December and April) was significantly higher compared to other dipterans. This model, based on weather parameters during the epidemic years, showed that *S. calcitrans* populations peaked in the months of LSD onset, in the studied farms. These observations and model predictions revealed a lower abundance of stable flies during October and November, when LSD affected adjacent grazing beef herds. Therefore, these findings suggest that *S. calcitrans* is a potential vector of LSD in Israeli dairy farms and that another vector is probably involved in LSDV transmission in grazing herds.	The vectorial capacity of *S. calcitrans* was determined solely by its abundance, the detection of LSDV in the captured vectors was not performed. The study relied on the assumption that vector seasonality remains approximately the same over the years. Data were based on the occurrence of LSD in each farm affected during the 2012- and 2013-outbreaks in Israel (i.e., retrospective data). The vector availability for those years was inferred under the assumption that vector seasonality remains approximately the same over the years. Nonetheless, the study had a good design with a long time period of dipteran trapping; models were appropriate, which gave sound conclusions that *S. calcitrans* was the potential vector of LSD in Israeli non-grazing dairy farms. However, it also implied that another vector could be the culprit for the outbreaks in beef grazing herds, but no vector was suggested.	Israel
[79]	Orynbayev et al. (2021)	ObD Vec.I. Ticks.	To describe the first cases of LSD in July 2016, in the Republic of Kazakhstan.	Blood and samples of internal organs (lymph nodes, spleen, lungs, skin with nodular lesions) were taken from sick and dead animals. Ticks, horse flies and biting flies from affected areas or dead animals were submitted to LSDV testing. PCR and gene sequencing were applied.	LSDV DNA was detected by PCR in all samples from dead animals and all ticks collected. Four *Dermacentor marginatus* and nine *Hyalomma asiaticum* ticks tested positive. LSDV DNA was also detected in three out of 21 horseflies (*Tabanus bromius*), and in one sample out of two *S. calcitrans* flies. The study concluded that the emergence of the disease coincided with a peak of vector activity; the introduction of LSDV in Kazakhstan was likely consecutive to the movements of infected livestock, with a subsequent transmission of the virus by blood-feeding insects.	The number of vectors sampled for the detection of the virus was very small, i.e., 13 ticks, 21 horse flies and 2 *Stomoxys* flies. The vectors potentially involved in the outbreaks could not be determined.	Kazakhstan
[80]	Makhahlela et al. (2022)	ObD Vec.I.	To increase the morphological and genetic information on the stable fly in South African feedlots, and to determine whether they may harbor LSDV and other pathogens of veterinary and economic importance.	This field study consisted in the sampling of stable flies from different feedlots across three South African provinces. Flies were identified according to the standard key morphological characters. PCR were performed to detect the presence of LSDV DNA.	LSDV DNA was detected in 8/53 samples, i.e., 15.08%. In South African feedlots, *S. calcitrans* harbours *A. marginale* and LSDV, which suggests that they may be involved in their mechanical transmission to livestock.	The study only shows that some stable flies were positive to LSDV in several south African feedlots. No other conclusion can be drawn from that study. No information is provided on how the sampling size was determined. Pool samples varied in terms of number of flies per pool. The authors did not specify in the results section if they were dealing with the number of pools or the number of insects positive to LSD. However, the study did show that flies positive to LSDV are present in South African feedlots.	South Africa
[12]	Issimov et al. (2020)	Exp. Vec.I.	To determine the vector competence of three *Stomoxys* spp. for the transmission of LSDV.	*S. calcitrans*, *S. sitiens* and *S. indica* were allowed to feed to repletion in experimentally infected-cows, after which they were tested for LSDV. Another batch was allowed to feed incompletely and then was moved to a healthy animal to complete feeding. PCR, serum neutralization test and virus isolation were performed to detect LSDV.	Recipient animals were all positive. *St. calcitrans*, *S. sitiens* and *S. indica* were negative 24 to 48 h post-feeding. All three species of flies demonstrated the capacity to ingest and harbor viral particles. They were able to transmit the virus within a 1 h time-interval between the meals. Moreover, LSDV was recovered from fly mouth parts within the same period and LSDV can survive in *Stomoxys* spp. at least 6 h following a meal on an infected animal. The mechanical transmission from infected to susceptible animals was demonstrated under laboratory conditions.	The study only determined the competence of the *Stomoxys* fly under laboratory conditions. See also the Same limitations as experimental study [8].	Not applicable
[13]	Issimov et al. (2021)	Exp. Vec.I.	The authors attempted to define the duration of LSDV retention in three *Stomoxys* spp., after intrathoracic inoculation, as well as virus potential to replicate after bypassing the midgut barrier.	A virulent LSDV strain was inoculated directly in the thorax (to bypass the midgut barrier) of adult flies of *S. calcitrans*, *S. sitiens* and *S. indica*. The flies were tested for the presence of LSDV DNA by gel-based PCR and virus isolation, at different times and days post- inoculation.	The virus was retained by the three *Stomoxys* spp., under laboratory conditions. LSDV was isolated from all three *Stomoxys* spp. up to 24 h post-inoculation while virus DNA was detectable up to 7 days post-inoculation. The outcomes illustrated the incompetence of *Stomoxys* spp. to serve as a biological vector of LSDV.	Although it demonstrated the incompetence of *Stomoxys* spp. as a biological vector and the virus was retained in the *Stomoxys*, the virus was directly inoculated into the thorax, which would this increases the probability of the fly to be positive to LSDV.	Not applicable
[14]	Paslaru et al. (2021)	Exp. Vec.I.	To investigate the role of *S. calcitrans* in the transmission of LSDV and its presence in four different farms in Switzerland.	Laboratory-reared *S. calcitrans* flies were exposed to LSDV-spiked blood. Engorged flies were incubated and body parts, i.e., heads thorax and abdomens, were tested for the presence of LSDV DNA for up to 72 h post-feeding. LSDV DNA was tested with a DNA mini commercial kit. Correspondingly, virus isolation in cell culture from regurgitated blood and in fecal samples of the flies was carried through. The presence of the fly in different farms and at high altitudes was assessed by trapping.	LSDV DNA was detected in heads, bodies, and regurgitated blood, up to 3 days post-feeding and up to 2 days post-feeding in the feces. Infectious virus was isolated from bodies and feces up to 2 days and up to 12 h post-feeding in the regurgitated blood. The viral load increased, which consolidates the role of *S*. *calcitrans* as a mechanical vector of LSDV. The fly was present in all farms investigated, including a farm located at 2128 m above sea level, showing that it is abundant and widespread.	Feeding of the stable fly was performed by placing them in cotton pads soaked with blood spiked with LSDV and not by placing them onto LSDV infected animals, which could increase the competence of the fly. Despite such fact, the experimental study showed that *S*. *calcitrans* was a competent mechanical vector of LSDV; its abundance in the farms showed that it would be a capable vector for spreading the virus between the animals.	Not applicable
[15]	Sohier et al. (2019)	Exp Vec.I.	To focus on the potential mechanical transmission of LSDV and to assess whether stable flies and horse flies could transmit LSDV when a shorter period between interrupted feeding on LSDV viraemic cattle followed by further feeding on naïve cattle would apply.	Bulls were experimentally infected. Three independent experiments were performed wherein biting flies, i.e., *S. calcitrans* and tabanids *Haematopota* spp., were allowed to feed for 10 min on LSDV infected-bulls (when animals were viremic or upon emergence of nodules). Potentially infected-insects were then allowed to feed for 10 min on susceptible cattle, one hour after the infective meal. In the other two experiments, insects were placed on the animals for two to three consecutive days. Blood was collected and biopsies of nodules were performed for RT- PCR analysis and virus neutralization test.	LSDV transmission by *S. calcitrans* was evidenced in the three independent experiments; LSDV transmission by *Haematopota* spp. was shown in one experiment. Results supported the mechanical transmission of the virus by these vectors. The study provided the first evidence of LSDV transmission by *S. calcitrans* and *Haematopota* spp. It is the first formal demonstration, under experimental conditions, that *S. calcitrans* is a vector of LSDV. LSDV was transferred from a donor to a receptive animal by flies exposed to the virus for maximum 3 days (and even 1 day for another animal) provides strong evidence that the transmission was mechanical and not biological. Horse flies also transmit LSDV, possibly more efficiently than stable flies. Indeed, one of the two horseflies put in contact with the receptive animal became positive. The large mouthparts of tabanids are helpful for mechanical transmission, as they can retain high blood volumes, and thus inoculate higher viral doses.	The competence of both stable and horse flies was determined. The capacity of both species was inferred by their vector characteristics and not by modelling. See also the same limitations as experimental study [8].	Not applicable
[92]	Sprygin et al. (2020)	LitRev Vec.I.	That literature review gained insight on the relationship between climatic conditions, ecological characteristics of the stable fly (*S. calcitrans* L.) and the observed spread of LSD across the Russian Federation, in 2015–2019.	Information on the entomology of *S. calcitrans* was compiled. Authors described the spread of LSD in cattle, in the Russian Federation, between 2015 and 2019; climatic conditions in the regions where the outbreaks occurred were recorded. The authors relied on data from domestic and foreign authors, on reports of Russian authorities on the spread of LSD in cattle and on meteorological data.	Data analysis showed that the activity of the stable fly mainly fits during the seasonal pattern of LSD outbreaks. However, some outbreaks occurred outside the activity period of the stable fly, pointing to other routes of transmission.	Vector capacity was based on previous studies.	Not applicable
[16]	Chihota et al. (2001)	Exp Vec.I.	Given that *Ae. Aegypti* was identified as an important vector of poxviruses, e.g., the myxoma virus, the study was undertaken to determine whether that mosquito species can act as an efficient mechanical vector of LSDV.	Fifty one week-old adult females of *Ae. Aegypti* fed on a lesion of experimentally infected steers. Transmission of the virus was then attempted by allowing these mosquitoes to feed on six susceptible cattle, at various times post- feeding. Transmission was confirmed by recording LSD clinical signs or recovering live virus from lesion material or blood of susceptible animals. DNA was extracted from infected mosquitoes and essayed by PCR. Cows were tested by PCR, virus isolation, virus neutralization index and their clinical score was recorded. The duration of virus transmission was also recorded.	Results showed that LSDV could be transmitted by *Ae.aegypti* for at least 6 days after infection. LSDV was able to survive in infected mosquitoes for at least 6 days, at a quite similar titer, and was then transmitted. The virus could be localized within the mosquito in a site protected from inactivation. The authors suggested a far more complex mode of transmission than a mere ‘dirty pin’. In conclusion, *Ae. Aegypti* female mosquitoes have the capacity to transmit LSDV mechanically, from infected to susceptible cattle. The clinical signs recorded in animals exposed to infected mosquitoes were generally mild, only one case being moderate. LSDV was long-suspected to be transmitted by insects, but these findings are the first to demonstrate that theory unequivocally; authors suggested that *Ae. Aegypti* was a competent vector.	Competence of the mosquito was determined by experimental infection. The main limitation is that mosquitoes were allowed to feed on a lesion, which is not necessarily the case in the field if one consider its anthropophilic behavior (i.e., preference to bite humans rather than animals).	Not applicable
[17]	Paslaru et al. (2022)	Exp Vec.I.	To expand on the findings of the insect ‘model vector species’. The LSDV suitability of mosquitoes and biting midges was investigated.	The mosquito species *Ae*. *Aegypti*, *Cx*. *Pipiens* and *Ae*. *Japonicus* were allowed infectious blood meals for 45 min. Field collected-*Culicoides* spp. and 2–3 day old laboratory reared-*C. nubeculosus* were exposed to an infectious blood meal for 30–45 min. The insects were tested for the presence of LSDV. DNA was extracted and isolated; bodies and head or wings were proxy for the virus dissemination at different time points after feeding.	Post-feeding viral retention lasted for 10 days for *Ae. Japonicas* and 7 days for *Cx. Pipiens*. In the three mosquito species investigated, more body samples where PCR-positive compared to head samples, indicating that the virus was not efficiently retained in the mouthparts and that there was no virus dissemination. Thus, mechanical transmission of LSDV by these species seems feasible in case of interrupted feeding. Viral DNA could be detected in feces of *Ae. Aegypti* until day 4 after feeding, although the significance of that finding is unclear. Thus, mosquitoes might serve as mechanical vectors of LSDV in case of interrupted blood meals. In *C. nubeculosus*, the virus was isolated from homogenized bodies up to the end of the experiment (10 days p.i.). Interestingly, Cq values decreased over time, and a disseminated infection at day 10 p.i. was identified in one insect. Considering the postulated absence of salivary gland barriers in *Culicoides* spp., these findings indicated that the laboratory-reared *C. nubeculosus* might behave as a biological vector of LSDV under laboratory conditions. LSDV did not persist in field-collected biting midges.	All insects were fed with LSDV-spiked blood meals and not directly on infected animals. Thus, competence may be inferred but the vectorial capacity of the mosquitoes cannot be implied. The virus was detected on homogenates of heads and body parts, rather than on the whole insects. Viable virus was isolated from homogenized bodies until day 10 post-infection. *Culicoides nubeculosus* was assumed as a biological vector, under experimental conditions, but based on a single insect with disseminated infection at day 10 post feeding, and the absence of salivary gland barriers in the *Culicoides* spp. The field-collected *C. nubeculosus* showed no persistence of LSDV, which suggests its most likely low competence.	Not applicable
[39]	Şevik and Doğan (2017)	ObC RiskF.	To determine the epidemiological status of observed LSD in several regions of Turkey; to evaluate the risk factors associated with LSDV infection; to determine the phylogenetic relatedness of the LSDVs circulating in Turkey; to assess the economic cost of LSD in surveyed regions; to investigate the potential role of *Culicoides* spp. in the transmission of LSDV.	Multiple samples were collected on dead animals: skin nodules, vesicle swabs, whole blood on EDTA tubes, lymph nodes, spleen, lungs, liver and heart; internal organs of aborted bovine foetuses were also sampled. *Culicoides* spp. were trapped in regions were the highest number of LSD cases was recorded. DNA was extracted and RT-PCR performed, along with sequence alignment and phylogenetic analysis. A questionnaire was submitted to livestock owners to collect information on LSD occurrence and other farm characteristics (location, type of herd, dairy of beef, total number of cattle on farm, number of cattle affected and dead from LSD, animal age, breeds affected and history of vaccination). Generalized linear mixed models investigated the risk factors influencing LSD prevalence.	The generalized linear mixed model provided the following results: European cattle breeds, small-sized family farms and farms located near a lake were identified as risk factors influencing LSD prevalence. The species of *Culicoides* in LSDV-positive pools was *C. punctatus*. The finding of LSDV in *C. punctatus* suggests that it may play a role in the transmission of LSDV. Furthermore, movements of infected animals to disease-free areas increase the risk of LSD introduction. Strategies of LSDV control should consider the risk factors identified in this study.	The model chosen to establish the factors influencing LSD prevalence was a linear model (not logistic), so it is hard to interpret the effect of a factor on LSD prevalence. Only LSD-suspected animals were sampled, and no sample size was calculated. The risk factors were not well established. Some factors assumed that the cattle died of LSD. The ‘near any lake’ factor is subjective as no distance from the farms affected by LSD was provided. *Culicoides* spp. were positive but the study inferences on their role in LSDV transmission were subjective.	Turkey
[81]	Sprygin et al. (2018)	ObD Vec.I.	To report the epidemiological investigation of an LSDV case caused by a vaccine-like strain in Russia, including attempts to detect the vaccine-like strain in several insect species trapped at outbreak location.	Samples of blood and scabs from cows of three affected farm (cows presenting clinical signs consistent with LSD) were collected and tested for field-LSDV DNA using a RT-PCR and vaccine-LSDV DNA using an assay developed for this specific work. An entomological surveillance based on insect trapping was implemented during 2 weeks after confirmation of the outbreaks. Trapped houseflies were divided into two batches for pooled and individual testing. The other captured insects, stable flies and lesser flies were tested individually. The testing was for the presence of LSDV DNA and vaccine-like LSDV DNA.	There was no evidence of field-LSDV strain circulation. The DNA of vaccine-LSDV was present in cattle. Stable flies tested individually, and to a lesser extent houseflies, were negative. The pool tested included three to five houseflies sampled randomly; 14 out of the 25 pools tested positive to vaccine-like LSDV DNA, but not to field-LSDV DNA. Flies were washed four times and tested. In *Musca domestica*, LSDV DNA was mainly detected in the first wash fluid, suggesting genome or even viral contamination on the insect cadaver. Internal contamination of insect bodies, without any differentiation between body locations, was also revealed; however, the clinical relevance for mechanical transmission is unknown. In this study, we discovered that *M. domestica* flies carried vaccine-like LSDV DNA whereas stable flies trapped at the same time were negative for both field- and vaccine-like LSDV DNA.	Although the first isolation of LSDV DNA from internal parts of non-biting insects is a very important finding, their role in LSDV transmission and spread still needs to be investigated.	Russia
[82]	Wang et al. (2022)	ObD Vec.I.	To investigate the first LSDV case caused by a vaccine-like strain at the western border of China; search for LSDV DNA in several insects captured around the region during the outbreak.	The authors implemented a surveillance of insects around the infected premises and the neighboring bordering areas. Insects were trapped; DNA was extracted and screened by RT-PCR and sequencing. A phylogenetic analysis was carried through.	The most abundant species captured during the campaign was *C. pipiens*, but all were negative to LSDV. It suggests that species was not involved in the LSDV epidemic. The overwhelming majority of captured insects were non-biting. Two kinds of non-biting flies, i.e., *Musca domestica* L and *Muscina stabulans*, were positive for vaccine-like LSDV. Despite such finding, there was no direct evidence to support cross-border transmission of the vaccine-like LSDV. The positivity of surface and negativity of internal contents indicated that non-biting flies could only acquire the virus by physical contamination.	The non-biting flies were the only insects to be positive to vaccine-like LSDV strain, and only on the surface of the body. Thus, their vectorial competence still needs to be determined.	China
[83]	El-Ansary et al. (2022)	ObD Ticks	To investigate and assess LSDV isolated from ticks collected in various outbreaks in Egyptian governorates and to characterize the virus at the molecular level.	Adult ticks were collected from cows in different Egyptian regions. Laboratory detection of LSDV was performed by PCR and sequencing. Further identification was carried on by non-serological methods.	*Rhipicephalus* (*Boophilus*) *annulatus* was the most prevalent tick species on cattle in the investigated regions; 15% of them were positive to LSDV. The majority of recent LSD outbreaks occurred in a period with mild and wet weather, i.e., from May to September, which favors tick activity.	The tick sample size was large, i.e., 4000 adult ticks. The number of positive samples was obtained by extrapolating the numbers of ticks from the positive pool samples wbich gave a total of 600 positive ticks out of 4000. Which extrapolated to 600 out 4000. Although it was a large sample size, the study only infers that ticks were positive to LSDV, which could determine their vectorial competence but not their capacity to transmit LSDV.	Egypt
[18]	Rouby et al. (2017)	Exp. Tick	To investigate the role of *R. annulatus* ticks collected from naturally infected animals in the transmission of LSDV.	Naturally infected cattle with LSD acute clinical signs underwent clinical examination. Samples of skin nodules and *R. annulatus* stages were collected from the sick cattle and examined by PCR; positive samples were confirmed by direct gene sequencing. Female engorged ticks were incubated for egg deposition; eggs and larvae that hatched were then screened for virus isolation and confirmed to be infected by PCR.	Detection of LSDV in tick larvae proved the possibility for these to be a potential source of infection for susceptible animals. The present study showed that females of naturally infected *R*. *annulatus* were able to transmit the virus vertically, via eggs to larvae. These findings suggest a high possibility for ticks to be a risk for the virus transmission and a field reservoir host of LSDV.	The competence of naturally infected ticks was established. Their role as a reservoir was not established, but only speculated.	Not applicable
[19]	Tuppurainen et al. (2015)	Exp. Tick	To investigate in vitro replication and/or survival of LSDV in cell lines derived from the tick species *R. appendiculatus*, *R. evertsi* and *R. (B.) decoloratus* and investigate the presence of the virus in live ticks collected from naturally infected cattle during LSD outbreaks in Egypt and South Africa.	LSDV was inoculated in tick cell lines: four semi-engorged female *Rhiphicephalus* spp. were collected in Egypt from three cows recovering from LSD but still showing some skin lesions and cabs. Tick samples were obtained from Egypt and South Africa. Detection of LSDV was carried out by real time PCR and virus titration.	There was no evidence of LSDV replication in tick cell lines, although the virus was remarkably stable, i.e., remaining viable for 35 days at 28 °C in tick cell cultures. Viral DNA was detected in two-thirds of the 56 field ticks. This is the first report to highlight the presence of potentially virulent LSDV in ticks sampled on naturally infected animals. All four ticks collected from Egypt were positive to LSDV. Out of the 52 samples collected from South Africa, 11 were *R. appendiculatus*, four *R. Boophilus*, seven *A. hebraeum*, four *H. truncatum*, two *Amblyomma* sp., six *Rhipicephalus Boophilus* sp.	The inability of LSDV to replicate in tick cell lines shed some information on the ability of the tick to act as a biological vector of LSDV.	Not applicable
[20]	Lubinga et al. (2014)	Exp. Tick	To further understand the role of ixodid ticks in the transmission of LSDV. The study aimed at determining the specific organs of adult *R. appendiculatus* and *A*. *hebraeum* infected by LSDV following an interrupted meal (intrastadial), and the transstadial persistence.	Nymphs and adult of *R. appendiculatus and A. hebraeum* ticks were orally infected by feeding on cattle infected experimentally by LSDV. For intrastadial infection, ticks were placed on infected animal for 4 days (on day 12 p.i.) after which they were collected for testing. LSDV was detected by immunohistochemistry, electron microscopy and RT-PCR. For transstadial persistence, nymphs fed on infected animals and once engorged, they were incubated for molting. Two months after emergence, they were put on LSD-free receptive animals and collected after for LSDV detection.	Intrastadial and transstadial transmissions were demonstrated for *R. appendiculatus*. The same observation had been performed for *A. hebraeum* in a previous study. The virus was able to cross the midgut wall and infect various organs, indicating a potential for biological development and transmission of LSDV by ticks. The salivary glands were the most affected organs, strengthening the previous report of LSDV occurrence in tick saliva.	Experimental infection affects the competence as it depends on the strain used and on direct feeding on an infected animal. A controlled environment facilitates infection, thus tick competence can be estimated. However, its vectorial capacity is still to be determined as these tick species do not spend their entire life cycle on the same host.	Not applicable
[21]	Lubinga et al. (2014)	Exp. Tick	To investigate the passage of LSDV from engorged *A. hebraeum* nymphs to adults, and from engorged female *R. decoloratus* to larvae, under cold temperatures, in order to determine their possible role in the overwintering of LSDV.	*A. hebraeum* and *R. decoloratus* female ticks were fed to repletion on LSD-free cattle. Thereafter, they were experimentally infected with LSDV on the day they dropped from the host. Nymphs were also infected and incubated at room temperature (25 °C), and at maximal and minimal winter temperatures, i.e., approximately 20 °C during the day and 5 °C at night. Virus isolation, RT-PCR and immunoperoxidase staining were performed to detect LSDV in the corresponding samples. Transmission electron microscopy was used in tick organs.	Transstadial and transovarial persistence of LSDV were observed in experimentally infected *A. hebraeum* nymphs and *R. decoloratus* females, after a 2 month-exposure to cold temperatures, i.e., 5 °C at night and 20 °C during the day. This finding suggests a possible overwintering of the virus in these tick species.	Same limitations as for study [20].	Not applicable
[22]	Lubinga et al. (2014)	Exp. Tick	To study the egg-transmission of LSDV from infected female ticks to the larvae in *A. hebraeum*, *R. appendiculatus* and *R. decoloratus*.	Laboratory infected cattle hosted adult *A. hebraeum*, *R. appendiculatus* and *R. decoloratus* during the viraemic stage. Two other animals were used as receptive hosts to assess the transmission of LSDV by *A. hebraeum* and *R. appendiculatus* larvae, respectively. Subsequently, these ticks fed on LSD-free animals to observe if mechanical transmission occurs.	The detection of LSDV in larvae of *A. hebraeum*, *R. decoloratus* and *R appendiculatus* indicates a transovarial passage of LSDV in these species. Authors showed LSDV transmission to receptive animals by *A*. *hebraeum*, *R. appendiculatus* larvae. These findings, in accordance with other studies, suggest a high possibility that ticks act as reservoir hosts of LSDV in the field. The overwintering in some tick species such as *R. decoloratus* may play a significant role in the overwintering of LSDV.	Same limitations as for study [20].	Not applicable
[23]	Lubinga et al. (2015)	Exp. Tick	To investigate the potential role of *Amblyoma hebraeum* ticks in mechanical/intrastadial and transstadial transmission of LSDV.	Adults and nymphs of *A. hebraeum* ticks were placed to feed on animals artificially infected with LSDV and subsequently transferred (nymphs after incubation up to 35 days to molt to adults) to naïve recipient cattle. Successful transmission of LSDV to recipient animals was determined through monitoring of clinical signs and laboratory detection of LSDV by RT-PCR, SNT and virus isolation.	This report provides further evidence of mechanical intrastadial and, for the first time transstadial, transmission of LSDV by *A. hebraeum*. These findings implicate *A*. *hebraeum* as a possible reservoir host in the epidemiology of the disease.	Same limitations as for study [20].	Not applicable
[24]	Tuppurainen et al. (2013)	Exp. Tick	To examine the potential for transovarial transmission of LSDV in *R. decoloratus* ticks.	Tick larvae were put on infected cows up to completion of life cycle and were allowed to lay eggs. After hatching, larvae were transferred to non-infected receptive cattle. Blood samples were collected from these cattle hosts at different days p.i. Laboratory detection of LSDV was performed by RT-PCR, SNT and virus isolation.	Receptive animals showed mild clinical signs with characteristic lesions. Thus, *R. decoloratus* ticks were able to transmit LSDV transovarially; this is the first report of such type of transmission for a poxvirus.	Same limitations as for study [20].	Not applicable
[25]	Tuppurainen et al. (2013)	Exp. Tick	To investigate if LSDV can be transmitted mechanically by African brown ear ticks *Rhipicephalus appendiculatus.*	Laboratory-bred *R. appendiculatus* males fed on experimentally infected viraemic cattle. Partially fed male ticks were then transferred on non-infected cows. The receptive animal did not develop any visible skin lesion post-infection.	The receptive animal became viraemic, showed mild clinical signs of LSD and seroconverted. Thus, *R. appendiculatus* ticks are able to act as mechanical vectors of LSDV. Additionally, *R. appendiculatus* males transmitted LSDV though feeding on visibly intact skin, which demonstrated that viraemic animals with no lesion at the tick-feeding site may be a source of infection. This is the first demonstration of poxvirus transmission by a tick species.	Same limitations as for study [20].	Not applicable
[26]	Lubinga et al. (2013)	Exp. Tick	To detect LSDV in saliva of *A. hebraeum* and *R. appendiculatus* adult ticks fed, as nymphs or adults, on LSDV-infected animals; thereby, the authors also aim at demonstrating transstadial or mechanical/intrastadial passage of the virus in these tick species.	Cattle were experimentally infected with LSDV and used to host nymphs and adult ticks of *A. hebraeum* and *R. appendiculatus*. The presence of LSDV in the saliva of these adult ticks was investigated by RT-PCR and virus isolation.	For the first time, LSDV was detected in the saliva of both *A. hebraeum* and *R. appendiculatus* ticks. At the same time, the authors demonstrated the persistence of LSDV in ticks between developmental stages (transstadial) and within the same stage (intrastadial) in both tick species.	Same limitations as for study [20].	Not applicable
[27]	Tuppurainen et al. (2011)	Exp. Tick	To investigate the potential role of ixodid (hard) ticks in the transmission of LSD.	Three common African tick species, i.e., *R. appendiculatus*, *A. hebraeum* and *R.* (*B.*) *decoloratus*, at different life stages, were fed on the skin lesion of infected animals during the viraemic stage. After feeding, the partially fed male ticks were transferred to the skin of non-infected “receptive” animals, while females were allowed to lay eggs; these eggs were tested by PCR and virus isolation. Nymphs were allowed to develop for 2–3 weeks before testing. The receptive cattle were tested for LSDV.	This is the first molecular evidence of potential LSDV transmission by ixodid ticks. The study evidenced transstadial and transovarial transmissions of LSDV by *R. (B.) decoloratus* ticks and mechanical or intrastadial transmission by *R. appendiculatus* and *A. hebraeum* ticks.	Same limitations as for study [20].	Not applicable
[73]	Kononov et al. (2019)	ObD Vac.	The present study follows up the epidemiological situation since 2016, and further examines samples containing vaccine-like LSDV strains, in the Privolzhsky Federal District, in 2017. That area is geospatially outside the zone affected in 2016 and where live vaccines against LSDV had never been authorized or knowingly used.	That field study investigated 13 out of 42 outbreaks. Whole blood, nasal swabs, and scabs were sampled and tested by PCR. Sequence analysis by amplifying the nucleotide sequences of RPO30 and GPCR gene to determine the type of strain of the LSDV.	Four outbreaks, i.e., two in backyard cattle and two in commercial farms were caused by vaccine-like LSDV strains, whereas the nine other outbreaks were attributed to field strains. Vaccine-like LSDV strains were isolated in two out of 21 backyard cattle and in 96 out of 2112 animals sampled in two commercial farms. Although live attenuated LSDV vaccines are prohibited in Russia, several vaccine-like LSDV strains were identified in the 2017 outbreaks, including commercial farms and backyard animals exhibiting clinical signs consistent with field LSDV strains. Sequence alignments of three vaccine-like LSDV strains showed a clear similarity to the corresponding RPO30 and GPCR gene sequences of vaccine attenuated viruses. How vaccine-like strains spread into Russian cattle remains to be clarified.	Not all outbreaks were sampled. The study managed to show that vaccine-like strains of LSD were the culprits of some outbreaks occurring in the region.	Russia
[74]	Aleksandr et al. (2020)	ObD Vac.	To report the emergence of a novel vaccine-like LSDV variant in Kurgan Oblast (Russia), along the southern Kazakh border, in 2018.	Samples of blood, serum and skin were collected from cows. DNA was extracted and RT-PCR performed to isolate the virus. Sequence and melt curve analysis were carried out as well.	Phylogenetic analysis of these additional loci placed the Kurgan/2018 strain in either vaccine or field groups, strongly suggesting a novel recombinant profile. This is another piece of evidence exposing the potential for recombination in capripoxviruses and the ignored danger of using live homologous vaccines against LSD. Authors discussed the need to revise the PCR-based strategy to differentiate infected from vaccinated animals and the potential scenarios of incursion. The contribution of KSGP/NI-2490-like strain to the emergence of the recently identified vaccine-like recombinant is discussed.	A new variant is described. That descriptive study accurately detected the vaccine-like strain.	Russia
[75]	Sprygin et al. (2020)	ObD Vac.	To perform full genome sequencing of the strain in order to characterize the genetic background of the strain responsible of an LSD outbreak that occurred during the winter 2019.	Field samples were collected, then the virus was isolated and cultured on lamb testis cells before purification. Genomic DNA was extracted and sequenced.	The proteins encoded by the ORFs are of high importance, since the findings show they mutated repeatedly from attenuated vaccine profiles to virulent wild-type profiles. Further work is needed to assess the extent to which recombinant vaccine-like strains spread in the country. Experimental work aimed at correlating the genetics of recombinant progeny with the virulence observed in infected hosts would also be interesting.	The study describes the importance of the proteins encoded by ORFs. This can provide indications on how recombinant vaccine-like LSDV strains spread in Russia.	Russia
[76]	Shumilova et al. (2022)	ObD Vac.	To report the detection and analysis of another recombinant strain from Saratov in 2019; that strain seems to be a clonal progeny of Russia/Saratov/2017, that overwintered in the region since 2017.	Viral samples were collected in the Saratov region, Russian Federation, in 2019. The samples were seeded on propagated and purified goat ovarian culture. DNA was extracted and sequenced.	The findings demonstrated the persistence of LSDV during winter and successful overwintering in a cold climate, which encourages additional research on LSDV biology.	No inferences were made on the origin of the vaccine strain. The reported outbreaks occurred in cold climates (i.e., outside the normal range of vector activity), which shows the overwintering of the virus. This conclusion is very important and the authors should have explained it more in details.	Russia
[77]	Sprygin et al. (2020)	ObD Vac.	To provide an overview of LSDV evolution in the Russian Federation since its first occurrence in North Caucasus in 2015 and further spread eastward, along the Kazakh border.	Blood samples were collected between 2015 and 2018 from cows presenting clinical signs of LSDV. DNA extraction was performed on 21 LSDV isolates from different regions and the presence of LSDV DNA was initially confirmed by PCR. Phylogenetic analysis was performed.	The findings showed that, between 2015 and 2018, the molecular epidemiology of LSDV in Russia split into two independent waves. The 2015–2016-epidemic was attributable to a field isolate, whereas the 2017-epidemic and even more the 2018-epidemic, were caused by novel importations of the virus, not genetically linked to the 2015–2016 field-strain. Such observations demonstrated a new emergence rather than the continuation of a field-type epidemic. Since recombinant vaccine-like LSDV isolates seem to have entrenched across the country border, the policy of using certain live vaccines requires revision as it is a clear biosecurity threat.	The study design describing the Russian LSD epidemic was well conducted. Inferences showed that new disease importations occurred in 2018. The authors could have provided hypothesis on the origin of emergence. Biosafety of the vaccine was questioned, but not the fact that it may have been poorly produced.	Russia
[56]	Byadovskaya et al. (2022)	ObC Vac.	To summarize the LSD outbreaks occurring between 2015 and 2020 across the Russian Federation and discuss the epidemiological features and possible risk factors in the current epidemiological situation.	Location data (i.e., geographical coordinates) of LSD outbreaks were collected, along with the date of the disease onset, the number of susceptible, infected and dead animals, average monthly temperatures and cattle density (2010 national statistics). A spatiotemporal analysis was performed, i.e., spatiotemporal clusters, a permutation model, a Poisson model and a directionality test.	The outbreaks of LSD occurred primarily in small holdings (backyard) rather than in commercial farms, mainly during the warm months, with the majority of outbreak peaks occurring in mid-summer. A highlight was made that in 2018 LSD cases continued until November and in snowy March 2019, i.e., winter conditions (snow and freezing temperatures) that preclude vector activity. Disease tended to form annual spatiotemporal clusters in 2016–2018, whereas in 2019 and 2020, such segregation was not evident.	The spatial-temporal analysis was well-conducted and gave a general picture of the clusters that occurred in Russia. Cold weather conditions, precluding vector activity, were highlighted. Although there were evident clusters, the effect of vaccination during the outbreaks was not mentioned neither included in the analysis.	Russia
[37]	Ma et al. (2021)	ObD Vac.	To characterize the genomic and phylogenetic features of an LSDV strain detected from cattle with typical LSD clinical signs in farms of southeast China.	Skin nodules, wounds, ocular, nasal, oral and rectal swabs were sampled from six affected cattle. The authors performed viral DNA detection, genomic sequencing and recombination analysis.	At least 25 putative recombination events between a vaccine strain and a field strain were identified in the genome of GD01/2020, which could affect the virulence and transmissibility of the virus. These results suggest that a virulent vaccine-recombinant LSDV, from an unknown origin, was introduced China, in Xinjiang, in 2019, and spread to Guangdong in 2020.	The study focused on the characterization of the LSDV strains detected in cattle clinically affected by LSDV. The question on how LSD was introduced in China remains unanswered.	China
[78]	Vandenbussche et al. (2022)	ObD Vac.	The aim of the study was twofold: (1) to analyze the composition of two batches of the Lumpivax^®^ vaccine and (2) to investigate a possible link between the vaccine and the recent vaccine-like recombinant LSDV strains.	The following processes were carried out: virus sequencing, reconstruction of vaccine strains, genome-wide analysis, recombination and breakpoint analysis.	The great divergence of recombinant strains in the batches (Neethling-like LSDV vaccine strain, KSGP-like LSDV vaccine strain and Sudan-like GTPV strain) suggests that they arose during seed production. The recent emergence of vaccine-like LSDV strains in large parts of Asia is, therefore, most likely the result of a spill over from animals vaccinated with the Lumpivax^®^ vaccine.	The study is well conducted and provides reasonable evidence that the vaccine-like strains causing the latest outbreaks in Russia and Asia are due to poorly manufactured Lumpivax^®^ vaccine.	Not applicable
[40]	Selim et al. (2021)	ObC RiskF.	To investigate LSDV seroprevalence in cattle with no history of vaccination, in some governorates of northern Egypt, and to assess the risk factors of infection.	Samples were collected randomly and classified according to the type of herd (dairy and beef), the breed (Baladi, mixed, and Holstein), the season (autumn, winter, spring, and summer), the age (range between <1 and >3 years old) and sex (male/female), if the sample came from animals with contact with other animals, water sources and feeding. Serum samples were analyzed by ELISA testing. The authors performed a multivariate logistic regression model and a chi-square analysis.	The multivariate logistic regression gave the following results. The risk of infection by LSDV was higher in Holstein breed, adult cattle and in the summer. Furthermore, communal grazing (i.e., sharing pastures) communal water points (i.e., shared water sources), introduction of new animal in a herd, and contact with other animals were identified as significant risk factors for the occurrence of LSDV infection in cattle.	The study was well designed and the risk factors well established. The only limitation of the study is that it relied on serology testing, and thus, the authors can only assume that the samples tested positive because of LSDV. Only unvaccinated cattle were assessed.	Egypt
[41]	Ince and Türk (2019)	ObC RiskF.	To analyze potential risk factors of LSD by a GIS and provide information to control its spread.	GIS systems and user interface programs were developed. The following data on LSD outbreaks were used: farms, cattle movements as well as temperature by the time of the outbreak. The authors assessed by combining an active disease follow-up, a questionnaire and retrospective data that focused on 70 pastoral and agro-pastoral farms, from August 2013 to December 2014. A multivariate logistic regression computed the strength of contribution of these risk factors to LSD occurrence.	The most significant risk factor affecting LSD prevalence was the proximity with the southern border of Turkey; the transmission of the disease to Turkey may have occurred from Syria and Iraq, since movements of live animals across the Syria–Iraq border exist and the first outbreak was recorded near the border. Analyses of morbidity risk factors of animal movements and animal markets showed that cattle purchased from other farms were at risk. For the transmission of LSD among farms, the most significant factor was cattle movements. LSD prevalence was significantly associated with purchasing infected animals that had not been tested or quarantined. The number of registered LSD outbreaks was higher in the summer, which suggests a seasonal distribution of LSD outbreaks during dry seasons. A seasonal trend of LSD outbreaks was observed in 2014. The number of reported outbreaks increased from June to October 2014, with a peak in August. The multivariate logistic regression concluded that cattle < 24 months old were more likely to be infected; females were more at risk than males and vaccinated animals were less at risk.	The risk factors were not well defined. The results of the final model were badly displayed and hard to interpret. Large confidence intervals show that there may be an issue in sample size or in the number of cattle tested. Conclusions were based on circumstantial evidence of movements across the Turkey-Syria border.	Turkey
[42]	Ochwo et al. (2019)	ObC RiskF.	To provide additional epidemiological information on LSD by estimating the herd and animal-level seroprevalence, and risk factors for seropositivity in herds with no history of vaccination, in the four major geographical regions of Uganda.	Blood was collected between July 2016 and August 2017, in Uganda districts; samples were screened by indirect ELISA for the presence of Abs against LSDV. The following herd characteristics were considered: cattle’s sex, age and breed, type of management, mean annual rainfall, region, contact with buffaloes, communal water source, newly introduced cattle, contact with wildlife and herd size. The authors applied multivariate logistic regression models.	The multivariate logistic regression model showed that pastoral and shared pastures, as well as fenced farms, were significantly associated with LSDV seropositivity. Other risk factors were: mean annual rainfalls of 1001–1200 mm and 1201–1400 mm, female cattle, age > 25 months and 13–24 months, and drinking from communal water sources.	Specific regions of Uganda were focused on in the study. This study relied on seroprevalence, by using an ELISA test to detect Abs against *Capripoxviruses*. It would have been useful to include the presence/absence of goats or sheep on and near the farms. Regarding the ‘communal’ water sources, the authors did not specify what they meant by ‘communal’. They did not explain either why they considered only herds with ≥ 20 cattle. The history of vaccination against LSD was included, which could give false positives to the serology test and over-estimate the prevalence, and finally the affect the results of the final model.	Uganda
[43]	Hailu et al. (2014)	ObC RiskF.	To estimate herd-level prevalence of LSD, and to assess the risk factors associated with the disease in Ethiopia; LSD is one of the major livestock disease problems in that country.	Questionnaires were carried out on affected Ethiopian farms between October 2012 and February 2013. The questionnaire was designed to ascertain the presence of LSD based on the farmer’s ability to recognize LSD clinical signs; it also gathered information on herd size, cattle age structure and management practices. The approach aimed at assessing the epidemiological factors associated with LSD in the previous two years. A multivariate logistic regression was carried out; the odds ratios of the potential risk factors of LSD occurrence were estimated.	The risk factors of LSD occurrence were: herd size (>22 animals), use of shared pastures and watering points, introduction of a new animal in the herd. Given that the characteristics of local management practices cannot be readily changed, disease control should rely on a greater use of effective LSD vaccines.	No sample size was determined. Herds were randomly selected but included in the study based on herd owner’s willingness to complete the questionnaire. The LSD status was determined by the farmer’s ability to recognize clinical signs associated with the disease. Although, the authors tried to account for it by recording commonly occurring skin diseases of cattle in the study areas: they were recorded from the district veterinary clinic for the differential diagnoses and by crosschecking whether the herd owner correctly related the disease event with the clinical signs of LSD. The possibility of error in detecting LSD signs or not would have affected the number of positive animals. Vaccination status was included.	Ethiopia
[44]	Issimov et al. (2022)	ObC RiskF.	To determine the prevalence of LSD, at individual and herd levels, and risk factors of LSD in West Kazakhstan.	The authors developed a questionnaire to assess the magnitude of LSD occurrence (based on the observation of clinical signs by the farmer) and associated risk factors. They considered herd size, breed, contact with other domestic animals, year and month of LSD occurrence and herd management (feeding and watering management, animal movement, vaccination, treatment). Multivariate logistic regression models were used to investigate the potential risk factors.	At animal level, the factors associated with LSD outbreaks included: medium and large herd size, purchase of animals and the sale of animals during an LSD outbreak. Herd management system had not altered after the outbreak. Therefore, the implementation of nationwide training programs is essential to improve the preparedness and awareness of farmers and veterinary personnel to control future emerging diseases.	The authors only considered farms located in west Kazakhstan. The categorization of farms, i.e., LSD-affected or not, relied on the presence or absence of LSD-affected animals in the farm. A farm was considered as affected if clinical signs characteristic of LSD were observed in at least one animal of the herd. This could have affected the number of true positives to LSD, as reporting the farm as positive or negative relied on the cattle holder’s observation only.	Kazakhstan
[45]	Gari et al. (2010)	ObC RiskF.	To address important knowledge gaps regarding the magnitude of LSD occurrence in different agro-climatic conditions and to identify associated risk factors.	The authors developed a questionnaire to gather the following information: year and month of LSD occurrence (LSD identified by the farmer), number, sex and age of affected animals that subsequently died, herd management (i.e., sedentary/transhumant farming system), herd size, vaccination against LSD, management of grazing/watering points, contacts with sheep and goats and introduction of new animals. The peak of biting fly activity (months) was observed and recorded. Data related to LSD occurrence in the study area and countrywide, as well as annual rainfall for the period 2000–2007 were registered as well. A multivariate logistic regression model was used, based on LSD occurrence at herd level.	The odds ratios of LSD occurrence in midland vs. highland, and in lowland vs. highland, were 3.86 (95% CI = 2.61–5.11) and 4.85 (95% CI = 2.59–7.1), respectively. A significantly higher risk of LSD occurrence was associated with communal grazing and watering management, as well as with the introduction of new cattle.	No sample size was calculated. The classify animals as LSD positive authors used farmers’ reports, reports from the district agricultural office documentation, and the national disease outbreak report database. All reporting systems were based on observations of clinical signs, The study used a crosschecking validation on clinical signs and described the disease to account for such bias. However, there is still the issue of confirming the true LSD status as it relied on the farmers’ ability to recognize clinical signs of LSD; the signs could be confounded with other co-morbidities. Vaccination status was not taken into account, which could have affected the risk factors.	Ethiopia
[46]	Odonchimeg et al. (2022)	ObC RiskF.	To investigate the current LSD outbreak in Mongolia to determine the prevalence and identify potentially associated risk factors.	The authors developed a questionnaire to gather the following information: general knowledge of LSD, herd’s proximity to water sources, vector activity, and water source, among others. Samples of suspected clinical cases were obtained. Cattle skin nodules were collected and submitted to PCR, virus isolation, DNA sequencing and histopathology. A phylogenetic analysis was also performed. Data were submitted to a multivariate logistic regression analysis.	In the multivariate model, females showed a significantly higher risk of LSD occurrence compared to males. On the contrary, adult animals, young cattle and locations near a tube well and pond (vs. near a river) were protecting factors.	The authors did not describe the study design. They did not calculate the sample size nor explained the sampling methodology (e.g., random, selected herd). The questionnaire was not described and the risk factor that were taken into account were not listed. Only factors which were significant in the univariate model. Only suspected clinical cases of LSD were sampled, thus could be an underestimation of cases. Locations of the farm near the tube well, pond or river, were used as risk factors but the distance classified as ‘near’ was never specified.	Mongolia
[47]	Hasib et al. (2021)	ObC RiskF.	To confirm LSD occurrence based on clinical, molecular and pathological identification and to unveil the plausible risk factors of LSDV infection in a region of Bangladesh.	The authors developed a questionnaire to collect demographic data on farms with suspected cases of LSD, i.e., breed, age, sex, and management practices such as source of water supply). A case was considered as LSD positive when an animal showed two or more defined clinical signs. Biopsy of nodular lesions was performed on sick or suspicious cattle, for confirmation; PCR, nucleotide sequencing and phylogenetic analysis were conducted on positive samples. Prevalence maps and multivariate logistic models were obtained.	A total of 19 farms, accounting a total of 3327 animals, were considered. Out of those, 120 were deemed as sick or suspected, and skin biopsies were collected from nodular lesions. The final multivariate model revealed that only foreign breeds and females were at higher risk.	Sampling was performed on suspect animals. Although no sampling size nor methodology were described, a large number of farms and animals were included in the study. Cattle were physically examined and farmers interviewed. Biopsy was taken only on suspect or clinically affected animals. No animal was considered as vaccinated, as it was a new outbreak in Bangladesh.	Bangladesh
[48]	Molla et al. (2018)	ObC RiskF.	To estimate the seroprevalence, to identify and quantify the risk factors contributing to the occurrence of LSD.	Sampling was performed in different regions. Antibody neutralization test detected Abs against LSDV. Herd level sensitivity and specificity were calculated. The variables included in the multivariate logistic regression model were: altitude (<2000/2000–2400/>2400 m above sea level), contact with other animals (yes/no), free animal movements (yes/no), presence of water bodies (river/pond/lake/damp swampy/irrigated lands) (yes/no), animal trade route in the study area (yes/no) and animal characteristics (breed, age and sex). Animals were categorized as calf (0.5–1 year), young (1–4 years old) and adult (≥4 years old); breeds were Holstein-Frisian cross and local Zebu.	A total of 2386 serum samples were collected. Generally, cattle population accounting many adults and that live in wet areas were at higher risk, whereas cattle in frequent contact with other cattle and other animal species had a lower risk, potentially due to a dilution effect of vectors. The final multivariate model identified the age as a risk factor, with animals aged 1–4 years old and ≥4 years were more at risk, compared to cows aged 6 months to 1 year old. Contacts with other animal species were protective. The presence of water bodies was a risk factor also.	The study focused on the central and north western parts of Ethiopia. The limitation of seroprevalence is that it cannot determine which *Capripoxvirus* causes the immune response. The authors did not consider the vaccination status in the analysis. The high number of serum samples ensured a robust estimation of the prevalence.	Ethiopia
[49]	Machado et al. (2019)	ObC RiskF.	To identify factors associated with 2014–2016 LSDV outbreaks and explore geographic areas at-risk, based on potential ecologically favorable conditions and the spatiotemporal dynamics of the disease.	Ecological niche modelling and fine spatiotemporally explicit Bayesian hierarchical model were applied to 2014–2016 LSDV outbreak data, from Middle Eastern ^(k)^, Central Asian ^(l)^ and Eastern European ^(m)^ countries. The outbreak database contained information on the geographical coordinates, date of occurrence, and numbers of susceptible and infected animals per herd.	Several independent variables influenced the spatiotemporal variability of LSDV. A risk was positively associated with precipitation and temperature, and negatively affected by wind. A contradiction and unresolved debate is the role of wind in the spread of the virus or via potential vectors, such as *S. calcitrans*. Authors found a negative effect of wind speed, i.e., the risk of LSDV would be reduced when winds are stronger. They also identified temperature as a factor increasing the relative risk of LSDV. Land cover may play a role in determining the risk.	The study covered a large geographic area, ignoring administrative boundaries, and instead, used a grid cell construction based on previous studies that estimated the distances over which LSDV could spread.	Middle East Central Europe and Asia
[50]	Alkhamis and VanderWaal (2016)	ObC RiskF.	To characterize the spatial-temporal dynamics of LSDV in Middle Eastern countries and to assess whether environmental and demographic variables could predict the geographic distribution of LSDV outbreaks reported in these countries between 2012 and 2015.	The authors used a maximum entropy ecological niche modelling method. They assessed multiple effective reproductive numbers to assess the transmission potential and efficacy of control and prevention measures during the epidemic that occurred in Middle Eastern ^(n)^ countries. Outbreak data from July 2012 to May 2015. The following environmental variables were included in the ecological niche model: climate variables, cattle/buffalo/sheep and goat density, global land cover and type ofl livestock production system. The following climatic variables were added to the model: monthly average, minimum and maximum temperatures, monthly rainfalls and altitude.	The most important environmental predictors that contributed to the ecological niche of LSDV included: annual rainfalls, land cover, average diurnal range temperature, type of livestock production system, and global livestock densities. Average monthly effective reproductive number (R-TD) was 2.2 (95% CI: 1.2–3.5), whereas the largest R-TD was estimated in Israel (R-TD = 22.2 (95% CI: 15.2–31.5) in September 2013, which indicated that the demographic and environmental conditions during this period were suitable to LSDV super-spreading events.	When using such approach to infer spatial patterns of infection risk, it is important to remember that there is no single ‘true’ model that predicts the risk across all contexts. Indeed, environmental factors contributing to the risk may differ across space and time. Authors did acknowledge that results might differ according to the input dataset. However, it also allowed the identification of spatial and environmental patterns that are consistent, regardless of the input dataset. The identified environmental predictors matched those identified in the literature, but it is important to consider that the resulting risk maps for LSDV occurrence are not definitive and need to be updated periodically as new data emerge. Thus, in the event of future epidemics, these analyses need to be repeated and refined in order to be subsequently used in surveillance, control, and prevention strategies.	Middle East ^(n)^
[51]	Ardestani et al. (2020)	ObC RiskF.	To assess the relationship between 2012 and 2016 LSDV outbreaks and environmental variables, in order to identify the most important environmental variables; to produce a distribution map of LSDV outbreaks in certain Iran areas, in order to determine at risk-areas based on potential ecologically-desirable conditions.	The authors used data on 2012–2016 LSDV outbreaks in Iranian provinces. For each LSDV outbreak, the database included information on its geographical coordinates (latitude and longitude), time data (month, season and year), social and political divisions of locations, type of herd, total number of farms, number of examined and affected animals and number of dead animals recorded. Ecological niche models were applied to data.	Rainfalls of the wettest period and the coldest season, as well as isothermality, were the bioclimatic variables explaining LSD prevalence. Coexistence of specific weather conditions, including defined humidity and temperature, is necessary for an LSD outbreak.	Although the authors present a fast and accurate approach to model the probability of LSDV, it is only worth for a specific area of Iran. Thus, inferences derived from this model need to be interpreted with caution.	Iran
[57]	Allepuz et al. (2019)	ObC RiskF.	To analyze and identify the association between the LSD outbreaks reported in Turkey, Russia, the Balkans and Israel, with climatic variables, land cover, and cattle density in order to predict the risk of LSD spread in neighboring free-countries of Europe and Central Asia.	The following data were added to the model: LSD outbreak locations, date of occurrence, geographical coordinates, animals at risk and animals clinically sick and dead. These data were gathered between July 2012 and December 2018 in the Balkans ^(o)^, Caucasus ^(o)^ and Middle East ^(o)^. The following variables, i.e., density of cattle, land cover and climate, were included in spatial regression models.	The results showed a significant effect of land cover on the occurrence of an LSD outbreak: areas at risk were mostly croplands, grassland, or shrub land. Cattle density, as well as areas with higher annual average temperature and higher diurnal range of temperatures, were also identified as risk factors.	Data used for this study relied mostly on passive reports of the veterinary services from the countries included in the analysis. The use of passive surveillance data has its limitations as cases or outbreaks could be underreported. This should be considered when interpreting the results.	Balkans Caucasus Middle East
[55]	Molla et al. (2017)	ObC RiskF.	To evaluate the spatial and temporal distribution of LSD outbreaks and to forecast future patterns of outbreaks in Ethiopia, based on data reported over the 2000–2015 period.	The authors used data of Ethiopian LSD outbreaks that occurred between the years 2000 and 2015. The records contained monthly information on place, time, and number of cases, deaths and animals at risk. The geographical distribution of LSD outbreaks over the 16 years was mapped, per administrative zone, using a geographic information system (GIS) software. The spread of the epidemic was also shown using SPMAP programs. Monthly average rainfalls for the period 1999–2013 were considered as well. Three seasons exist in Ethiopia (a) February to May, (b) June to September and (c) October to January, which registers the highest rainfall. Time series analysis and spectral analysis were conducted to detect seasonality and cyclical patterns in the LSD outbreak time series.	The highest LSD incidences were registered in warm and humid highlands, while the lowest occurred in hot and dry lowland areas. The regions receiving relatively high rainfalls for a reasonable period are conducive to the replication and survival of blood-feeding arthropods and thus, to the spread of the disease. The occurrence of LSD outbreaks was seasonal, with a peak registered in October and the lowest number in May and at the end of the long rainy season. Additionally, LSD outbreaks do not occur at random over time: authors demonstrated the seasonality by spectral analysis. The seasonal variation of LSD outbreaks might be related to the variation in temperatures and rainfalls between seasons, leading to variable arthropod densities in the environment.	The presence of a long-term trend or season effect was determined only by simple examination of the graph, no statistical analysis was conducted to assess statistical significance. The existence of al long-term trend in LSD outbreaks was modelled by linear regression and using the number of LSD outbreaks (or trend component of the outbreak). Authors establish the limitations of the model that does not consider the correlation between successive values of the time series. This means one can only gain advantage of using short-term forecasts. Additionally, the wide confidence interval indicates the need of frequent updating of the model by incorporating the latest outbreak reports.	Ethiopia
[53]	Molla et al. (2017)	ObC RiskF.	To better understand the dynamics of LSDV outbreaks and to quantify transmission rate and reproductive ratio (R0) between animals.	The transmission parameters relied on a susceptible-infectious recovered (SIR) epidemic model with environmental transmission, and estimated using generalized linear models.	The survival rate of infectious virus in the environment equaled 0.325 per day, based on the best-fitting statistical model. The daily transmission rate between animals reached 0.071 (95% CI = 0.068–0.076) in the crop-livestock production system and 0.076 in the intensive production system (95% CI = 0.068–0.085). The R0 of LSD between animals was 1.07 in the crop-livestock production system and 1.09 in the intensive production system. These R0’s provides a baseline to assess the efficacy of various control options.	The daily transmission rates of crop livestock systems and intensive systems did not differ significantly. That suggests that the knowledge of these parameters alone is not sufficient to predict the risk of LSD in the different production systems.	Ethiopia
[54]	Mercier et al. (2018)	ObC RiskF.	To estimate the LSDV spread rate for a further use in risk analysis of LSDV introduction in other European countries.	LSD outbreaks were mapped according to their geographical coordinates. Study time period ranged from the date of the first occurrence, in May 2015 (western Turkey), to August 2016. Outbreak mapping and thin plate spline regression models were used.	The frequency of outbreaks was highly seasonal, with little or no transmission in the winter period. The skewed distribution of spread rates suggested two distinct underlying epidemiological processes, i.e., (i) local and distant spread possibly related to vectors and (ii) cattle trade movements. Low spread rates were probably related to local LSDV transmission by infected arthropods and contacts between infected and naïve cattle, covering small daily distances. On the other hand, high spread rates might be related to the movements of infected animals between farms trade, to/from cattle markets or to slaughterhouses.	This analysis considered only the outbreaks reported up to the end of August 2016, and did not include all Albanian outbreaks; 2323 out of 3585 outbreaks occurred after this date. In addition, the analysis implicitly includes the impact of stamping out infected herds on the rate of spread, which was implemented in all affected countries except Albania. Although unavoidable, the maximum spread rate due to possible under- or delayed reporting is probably unstable. Vaccination campaigns must have strongly influenced the spread of the disease and vaccination data were not incorporated in the model.	Balkans
[60]	Gubbins et al. (2020)	ObC RiskF.	To explore how the force of infection depends on the distance between non-infected and infected herds, to assess evidence for seasonality in the force of infection and to estimate the impact of vaccination on the spread of LSDV.	The authors used LSD outbreak data from Albania collected in 2016. A kernel-based approach described the transmission of LSDV between herds. In this approach, all transmission routes were combined in a single generic mechanism with the probability of transmission from an infected to a non-infected herd assumed to depend on the distance between them (i.e., the transmission kernel).	It was shown that most of the transmission occurred over short distances (<5 km), but with an appreciable probability of transmission over longer distances. The authors evidenced a seasonal variation in the force of infection associated with temperature, possibly through its influence on the relative abundance of the stable fly *S. calcitrans*. Both results are consistent with a transmission of LSDV by the bites of blood-feeding insects, though further work is required to incriminate the vector species.	The approach of combining all transmission routes into a single generic mechanism, and the assumption of susceptibility of an uninfected herd and the infectiousness of an infected herd to be both proportional to the number of cattle in the herd, could affect the kernel shape.	Albania
[58]	Punyapornwithaya et al. (2022)	ObC RiskF.	To determine the spatio-temporal patterns of LSD outbreaks in dairy farms, in northeastern Thailand, in order to better understand the epidemiology of LSD outbreaks affecting dairy farms.	An LSD case was defined as a dairy cow displaying LSD clinical signs. Blood was sampled to confirm an infection by LSDV. The following epidemiological data were collected: number of dairy cattle with LSD clinical signs, deaths with clinical signs and the number of all dairy cattle on the farms. The geographical coordinates of each farm were recorded. A spatio-temporal analysis using space-time permutation models, Poisson and Bernoulli models was performed.	The authors concluded that, because there are few cattle movements between dairy farms, the spread of LSD was less likely due to close contacts between cattle from different farms. Furthermore, the spread of LSD was likely caused by insect vectors, which are abundant in most dairy farms in Thailand. Indeed, the finding that LSD outbreaks were located in a large number of farms and over a short period, and that several farms were concentrated in the area, suggests that LSDV was probably transmitted by insect vectors.	The authors did not draw any direct conclusion from the model regarding the vector transmission. Spatial temporal patterns showed that several farms concentrated on the same were affected over a short period of time. This with the fact that there were few cattle movements among farms made authors reach the conclusion that it the spread was attributed to insect vectors.	Thailand
[61]	Klausner et al. (2017)	ObC RiskF.	To examine the possibility of LSDV introduction in Israel, in 1989 and 2006, by long-distance wind-associated movements of infected vectors from Egypt.	Israeli outbreaks were reported in August 1989 and on 7 June 2006. Backwards Lagrangian trajectories (BLTs) analysis was conducted. It consists in reconstructing the travelling path of an air parcel from its source to a given receptor. These trajectories are calculated using the re-analysis of available meteorological fields as inputs. Synoptic systems climatologically associated with the period preceding the outbreaks were identified, along with typical atmospheric transport routes during the synoptic systems. Three-dimensional backwards Lagrangian trajectories (BLTs) were calculated using the hybrid single-particle Lagrangian integrated trajectory model.	At the first stage, the relevant synoptic systems that allowed wind transport from Egypt to Israel during the 3 months preceding each outbreak were identified. The analysis revealed several events in which atmospheric connection routes between the affected locations in Egypt and Israel were established. Specifically, in 1989, Damietta and Port Said stand out as likely sources for the outbreak in Israel. In 2006, different locations acted simultaneously as potential sources of Israeli outbreak. The analysis pointed out Sharav low and Shallow Cyprus, low to the North, to be the most likely systems to enable windborne transport from Egypt to Israel. These findings are of high importance to analyze the risk of transmission of vector-borne viruses in the eastern Mediterranean region.	The study only considered Israel. The difficulty in conducting such type of analysis stems from the uncertainty regarding the exact arrival of the virus on the receptor site (in this case, Israel). Although authors concluded that winds could have carried the infected vector from Egypt to Israel, the vectors (in this case *Stomoxys*) competency was not mentioned, and thus atmospheric travel under dry conditions is possible but not ideal for the survival of the flies. Hence, a doubt remains on the viability of such route of spread.	Israel
[84]	Horigan et al. (2018)	Risk A. QL	The qualitative assessment focused on the probability of LSDV introduction in the UK, between June 2017 and June 2018, and the probability of onward transmission in the country.	A qualitative risk assessment was conducted. The approach was based on the framework set out by the OIE. The risk questions to be addressed were: (a) what is the probability of introduction of LSDV in the UK within the next year? (b) what is the probability of onward transmission of LSDV in the UK; could it be introduced within the next year? The following risk pathways of introduction were considered: infected live animals legally/illegally imported in the UK, contaminated animal products legally/illegally imported and infected vector imported to the UK.	The overall risk of potential introduction and further onward transmission of LSDV was “very low” through livestock, but with a “high” probability of onward transmission. The risk of introduction was considered ‘very low’ via vectors, but the probability of onward transmission was ‘high’. Exotic animals, germplasm, hides/skins, meat and milk products were negligible for both probabilities.	The study conducted the risk assessment of entry using and describing the correct guidelines. As any other qualitative risk assessments, it depends on the knowledge of the experts who conducted the categorization.	United Kingdom
[85]	Gale et al. (2016)	Risk A. QL	A qualitative assessment of the risk of importation of one infected product (i.e., skin/hide or bale of wool) through legal trade into the UK.	A qualitative risk assessment was conducted. The approach relied on the framework set out by the OIE. The specific risk question was: what is the probability that a whole skin/hide or bale of wool legally imported from a European Union Member State (MS) experiencing an ongoing outbreak is infected with capripoxvirus at the point of entry into the UK?	The predicted risk of importation of LSD virus per cattle hide/skin was also low (assuming LSD was to emerge in a EU MS with similar herd prevalence to sheep and goat pox in 2013/14 in Greece). The amount of LSDV on an infected cow hide, if imported, may be very low. It is recommended to recalculate the risks of entry for capripoxviruses if outbreaks occur elsewhere within the EU.	The risk assessment used the correct guidelines. Given that only EU Member States were considered in the analysis, the risk assessment is most likely to give a low risk.	United Kingdom
[86]	Farra et al. (2021)	Risk A. QL	A qualitative risk assessment was conducted, with the aim (i) to investigate the probability of LSDV introduction in Ukraine and, (ii) if introduced, the probability of onward transmission in the country within the next year.	A qualitative risk assessment was conducted. The approach relied on the framework set out by the OIE Handbook on Import risk analysis. The overall questions of the risk assessment were: (a) probability for LSDV to be introduced in Ukraine within the next year; (b) if LSDV is introduced in Ukraine, probability of onward transmission in the country within the next year; (c) risk pathways, i.e., cattle, wild ruminants, semen, embryos, biomaterials, skin, hides, trophies, meat, milk and vectors.	The illegal trade of cattle was considered the highest risk of LSD introduction. However, the probability was estimated to be low. When assessing the probability of an animal to be exposed to the virus and responsible for the further transmission in Ukraine, a high probability was estimated for flying vectors.	The risk assessment was very complete in using all the risk pathways with the right guideline. The study was described very well. The limitations are similar to any qualitative risk assessment, i.e., it relies on the knowledge of experts.	Ukraine
[89]	Saegerman et al. (2018)	Risk A. QT	In order to estimate, for France, the threat of introduction of vectors through animal trucks (cattle or horses) coming from at-risk countries (Balkans and neighboring countries), a quantitative import risk analysis (QIRA) model was developed according to the international standard.	The authors used a stochastic model to assess the probability of importing cattle from an at-risk area, that can be infected with LSDV before its detection. They also estimated the probability that trucks come from an infected farm located in the at-risk area and the probability of an animal to be infected already in the farm but without clinical signs. The authors also considered the probability of the virus surviving in *Stomoxys* spp. and the probability that *Stomoxys* spp. would survive during transport (survival of the fly was estimated at 2–3 days).	The authors used stochastic QIRA modelling and combined experimental/field data and expert opinion. The yearly risk of LSDV being introduced by stable flies (*S. calcitrans*) travelling in animal trucks was between 6 × 10^−5^ and 5.93 × 10^−3^ with a median value of 89.9 × 10^−5^; it was mainly due to the risk related to insects entering farms in France from vehicles transporting cattle from the at-risk area. The risk related to the transport of cattle going to slaughterhouses or the transport of horses was much lower (between 2 × 10^−7^ and 3.73 × 10^−5^ and between 5 × 10^−10^ and 3.95 × 10^−8^ for cattle and horses, respectively). The disinsection of trucks transporting live animals is important to reduce this risk.	Authors mentioned the limitation of the QIRA modelling which were related to the choice of assumptions and worst case scenarios (proportion of infected *Stomoxys* equivalent to the proportion of contagious cattle, absence of cleaning, disinfection and disinsectisation of the truck used for the transport of animals, absence of unloading of animals during transport, only *Stomoxys calcitrans* considered as mechanical vector of LSDV, proportion of mixed cattle and equine activities in countries of origin unknown and consequently estimated at the same as in France, and probability of infecting cattle on the destination farm of 100%.	France
[88]	ANSES, 2017	Risk A. QL	To assess the risk of LSD introduction in France.	The authors assessed the risk of LSD introduction in France taking into account the different risk factors of introduction; The probability was ‘only’ for the probability of a first LSD outbreak on the French territory for the specific year of when the study was conducted and it was based on the epidemiological situation of LSD in January 2017, according to the exiting European regulations at that date and using trade data of the year 2016. An assessment of the risk of a first LSD outbreak in France was performed, depending on the different virus sources and their possible ways of introduction (live animals and their products—semen and embryos, vectors, inert media, etc.). The risk assessment was carried out according to a quantitative approach for the introduction pathways considered by the experts as most likely (movements of animals, movements of arthropod vectors,); in the other cases, the approach was qualitative.	Only animals from EU at-risk areas (MS that reported outbreaks) were taken into account in the analysis. The probability of LSD introduction by live animals was limited to the risk of introduction by live cattle. The quantitative probability of a first LSD outbreak in France following the introduction of infected live cattle was estimated between 0.004% and 0.32% (95% CI), which corresponds to an ‘extremely low to low’ qualitative probability (3 to 5 on AFSSA 2008 scale, which ranges from 0 to 9). The probability of a first LSD outbreak in France following the introduction of infected live cattle for the slaughterhouse is therefore estimated to be null. The risk of LSD introduction by long-distance road transports of vectors is limited to the risk of introduction by *Stomoxys* spp. The quantitative probability of a first LSD outbreak in France following the introduction of infective vectors transported with live cattle was therefore estimated between 0.002% and 0.44% (95% CI), which corresponds to an ‘extremely low to low’ qualitative probability (3 to 5 on AFSSA scale). The probability of introduction via other modes was considered as null.	The qualitative risk assessment was very thorough. Not only experts’ opinion was used but also quantitative data regarding cattle and horse entering France, which gave a more certain assessment.	France
[90]	Saegerman et al. (2019)	Risk A. QT	To assess the risk of LSD introduction through cattle imports.	In order to estimate the threat for France, a QIRA model was developed to assess the risk of LSD introduction in France through cattle imports.	Based on available information, and using a stochastic model, the probability of a first LSD outbreak in France, following the import of batches of infected live cattle for breeding or fattening, was estimated at 5.4 × 10^−4^ (95% probability interval [PI]: 0.4 × 10^−4^; 28.7 × 10^−4^) in summer months (during high vector activity) and 1.8 × 10^−4^ (95% PI: 0.14 × 10^−4^; 15 × 10^−4^) in the winter.	The QIRA model depends on the available data and information on live-animal trade between European countries other than France (in particular between infected countries and countries bordering France) was not available for the French experts.	France
[87]	Ince et al. (2016)	Risk A. QL	To assess the epidemiology of LSD, its transmission mechanisms, and the potential role of risk factors. Qualitative estimates of the risk, spatial variation in risk, and the factors associated with the risk of LSD introduction and spread in animal markets are a prerequisite for developing specific policies to prevent or control epidemics.	The authors performed a qualitative risk assessment. The approach relied on the framework set out by the OIE Handbook on Import risk analysis. The risk question was the probability of cattle with LSD being introduced to the animal market? The farms with reported outbreaks were observed by a veterinarian, who examined any suspect animal. The risk estimation and management were carried out. Two risk pathways were identified, i.e., (1) probability of cattle to be exposed to LSDV from seasonal migration, and (2) probability of exposing cattle to LSD through veterinary equipment.	The risk (probability) of a farm being infected was estimated as ‘medium to high’, such as the risk (probability) of an animal being infected on a farm. The risk (probability) of not detecting LSD in non-certified and infected cattle was ‘high, such as the risk (probability) of LSD introduction to non-infected provinces through animal movements and the risk (probability) of cattle to be exposed to LSDV from seasonal migration. Finally, the risk (probability) of exposing cattle to LSD through veterinary equipment was estimated as ‘medium’.	The release assessment categories were not clearly detailed in the results. The same limitation as above, regarding qualitative risk assessments, apply.	Turkey
[91]	Taylor et al. (2019)	Risk A. QT	To provide a generic framework for quantitative risk assessment of disease introduction using LSD as a case study.	The authors created a generic framework, i.e., they defined the risk of infection as the probability of one or more initial infections in the native susceptible population in a specific area. Then the framework was applied to a single pathway using LSD as a case study (2016-outbreak in the Balkans). The risk assessment was performed on three spatial scales, i.e., countries, regions and individual farms.	Croatia (assuming no vaccination occurred) had the highest mean probability of infection, beating out Italy, Hungary and Spain. The detection of infected cattle at importation does reduces the risk, but proportionally lower for countries with the highest risk. The results were consistent across the spatial scales, while in addition, at the finer spatial scales, specific areas or individual locations on which to focus surveillance were identified.	Only a single pathway of introduction was used, i.e., the number of cattle traded within the EU, and on the basis of LSD prevalence in the country of origin of cattle. Thus, results are conditioned by the prevalence of LSD in the EU.	Europe

Legend: ^(a)^ LSD = Lumpy skin disease; ^(b)^ LSDV = Lumpy skin disease virus; ^(c)^ ELISA = Enzyme-Linked Immunosorbent Assay; ^(d)^ SNT = serum neutralization test; ^(e)^ RVF = Rift valley fever; ^(f)^ RT-PCR = Real-time polymerase chain reaction; ^(g)^ PCR = Polymerase chain reaction (PCR); ^(h)^ R0 = basic reproduction number; ^(i)^ p.i. = post-infection; ^(k)^ Middle Eastern countries: Iraq, Iran, Turkey; ^(l)^ Central Asian countries: Kazakhstan; ^(m)^ Eastern European: Albania, Azerbaijan, Bulgaria, Georgia, Greece, Macedonia, Montenegro, Russia and Serbia; ^(n)^ Middle Eastern countries: Iran, Turkey, Syria, Lebanon, Israel, Palestinian Territories, Jordan, Iraq, Egypt, Libya, Sudan, Djibouti, Eritrea, Somalia, and countries of the Arabian Peninsula (Saudi Arabia, Yemen, Oman, United Arab Emirates, Qatar, Bahrain, Kuwait, Azerbaijan and Cyprus; ^(o)^ Balkans, Caucasus and Middle East countries: Albania, Bulgaria, Cyprus island, Egypt, Former Yugoslav Republic of Macedonia, Georgia, Greece, Iran, Iraq, Israel, Jordan, Kazakhstan, Kosovo, Kuwait, Lebanon, Montenegro, Russian Federation, Saudi Arabia, Serbia, Turkey and West Bank. **Nomenclature**: **ObD** = Observational descriptive study; **ObC** = Observational cross sectional study; **Exp** = Experimental study; **Lit.Rev.** = literature review; **Host** = main objective to determine host of LSDV; **R.T.** = Study investigating LSD main routes of transmission; **S.T.** = Study investigating the seminal transmission of LSD; **I.U.** = Study investigating intrauterine transmission; **Vec.I.** = Main objective to determine insect vectors of LSDV; **Tick** = main objective to investigate the role of ticks as LSDV vectors; **Vac** = study investigating the role of vaccines in LSD outbreaks; **RiskF** = main objective was to identify the main risk factors for an LSD outbreak; **RiskA** = Risk assessment study; **QL** = Qualitative; **QT** = Quantitative.
